# Anisotropic Porous Iron-Based Nanoparticles through Two-Step Hydrothermal and Hydrogen-Based Reduction: Enhanced Magnetic Performance for Potential Biomedical Applications

**DOI:** 10.1021/acsami.4c21063

**Published:** 2025-03-07

**Authors:** Sofia Caspani, Francisco Javier Fernández-Alonso, Sofia M. Gonçalves, Celia Martín-Morales, Belén Cortes-Llanos, Bruno J. C. Vieira, João Carlos Bentes Waerenborgh, Laura C. J. Pereira, Arlete Apolinario, João P. Araújo, Maria Victoria Gómez-Gaviro, Vicente Torres-Costa, Miguel Jose Manso Silván, Célia Tavares de Sousa

**Affiliations:** † IFIMUP - Departamento de Física e Astronomia da Faculdade Ciências da, 26706 Universidade do Porto, Rua do Campo Alegre 1021 1055, Porto 4169-007, Portugal; ‡ Departamento de Física Aplicada, 16722 Universidad Autonoma de Madrid, Ciudad Universitaria de Cantoblanco, Madrid 28049, Spain; § Centro de Microanálisis de Materiales, Universidad Autónoma de Madrid (UAM), Campus de Cantoblanco, Madrid 28049, Spain; ∥ Centro de Ciências e Tecnologias Nucleares, DECN, Instituto Superior Técnico, Universidade de Lisboa, Bobadela 2695-066, Portugal; ⊥ IiSGM, Instituto de Investigación Sanitaria Gregorio Marañón, Madrid 28007, Spain

**Keywords:** hydrothermal, iron-based nanoparticles, anisotropy, chemical reduction, biocompatibility

## Abstract

Iron-based nanoparticles have emerged as promising candidates for diverse biomedical applications, including cell separation, targeted drug delivery, hyperthermia therapy, and magnetic resonance imaging. This study reports the scalable synthesis of high-magnetization iron-based nanoparticles with controlled anisotropic shapes, achieved via a two-step process. Hematite nanoparticles, featuring nanocube, nanoellipse, and nanoneedle morphologies, were synthesized through the hydrolysis of ferric chloride in the presence of ammonium dihydrogen phosphate, with the morphology precisely tuned by adjusting reagent concentrations. These hematite nanoparticles were subsequently reduced in a hydrogen-based direct reduction at 480 °C, yielding iron-magnetite nanocomposites that retained their anisotropic shapes, exhibited significant porosity, and achieved an exceptional saturation magnetization of 207 emu/g - approximately 150% higher than conventional magnetite nanoparticles. Comprehensive characterization via SQUID magnetometry, Mössbauer spectroscopy, Rietveld refinement of X-ray diffraction data, and XPS for surface analysis confirmed the formation of metallic iron nanoparticles covered by a magnetite shell. Biocompatibility studies demonstrated the biocompatibility of these nanoparticles across a wide concentration range, underscoring their suitability for biomedical applications.

## Graphical abstract

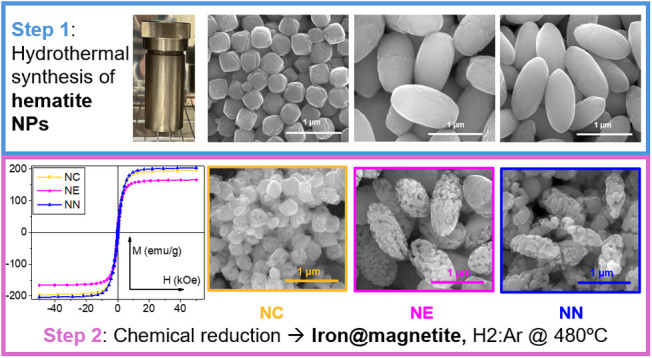

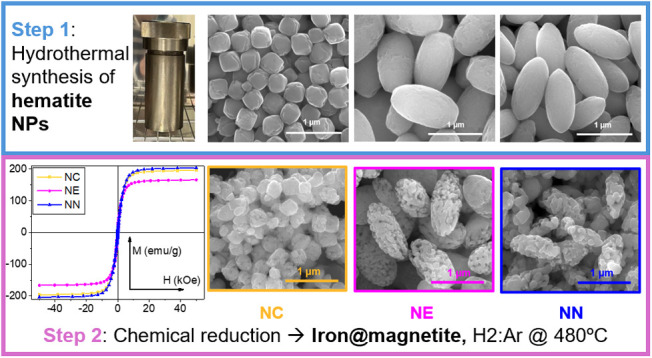

## 1 Introduction

Over the past decades, magnetic nanoparticles, particularly iron oxide nanoparticles (IONPs), have garnered significant attention across various scientific fields.^1–3^ In particular, IONPs have been extensively studied in biomedicine due to their unique magnetic properties, large influence of an external magnetic field, and ultimately high biocompatibility.^1,3^ Therefore, IONPs demonstrate immense potential for a range of applications, biomedical and pharmaceutical, including drug delivery, magnetic separation, magnetic resonance imaging (MRI), and hyperthermia-based cancer treatment,^2,4,5^ among others. In such therapies, nanoparticles must exhibit stability, biocompatibility, and distinct magnetic properties, including high magnetic response, remanence, high energy product, elevated saturation magnetization (Ms), and significant coercivity.^1,4,6^ Control over key parameters such as morphology, porosity, and structural features is particularly important for achieving desired magnetic properties, which can be accomplished by fine-tuning the chemical composition, shape, and size of the nanostructures. These modifications not only enhance their responsiveness to external magnetic fields but also ensure their biocompatibility and effectiveness in biomedical applications.^3,4,7,8^

Therefore, IONPs in various crystalline polymorphs have garnered significant interest due to their nanoscale form. Magnetite (Fe_3_O_4_), maghemite (γ-Fe_2_O_3_), and hematite (α-Fe_2_O_3_) are the primary forms of iron oxides, differentiated by their oxygen anion arrangements and the positioning of iron cations in tetrahedral or octahedral sites. Hematite exhibits weak ferromagnetic behavior, while magnetite and maghemite demonstrate strong magnetic responses under an external magnetic field. However, maghemite is thermally unstable and can transform into hematite at elevated temperatures.^9^ Among these, magnetite stands out as the most suitable form of iron oxide for nanoparticles, requiring precise control of its magnetic properties for effective use in biomedical, pharmaceutical, and other magnetically driven applications. While magnetite provides better intrinsic stability despite lower magnetic performance, iron-based nanoparticles offer superior magnetic properties (higher Ms and Mr) but require protective coatings for stability. Recent research focuses on Fe–Fe oxide nanoparticles to synergize the strengths of both materials—combining magnetic performance, stability, and resistance to aggregation while controlling the shape morphology, being this combination a challenge.^8,10,11^

Spherical-shaped superparamagnetic iron oxide nanoparticles (SPIONs) are commonly used, but their small size (<20 nm^12^) restricted by the superparamagnetic regime and low magnetic moments limit their effectiveness.^12,13^ To compensate for the reduced saturation, higher concentrations are often required, which can result in harmful effects in terms of toxicity levels. Conversely, at lower concentrations, SPIONs lose targeting efficiency and may become toxic by diffusing into cell membranes and damaging intracellular components.^12,14^ To overcome these limitations, controlling and tuning the SPIONs’ morphology enables precise control of magnetic properties. For example, cubic-shaped IONPs demonstrated enhanced SAR (specific absorption rate) values suitable for hyperthermia treatment.^5^ Comparative studies revealed superior SAR in cubic and octahedral shapes of nanoparticles,^10,15^ as well as nanorods, compared to spherical counterparts.^16^

Precise control over the synthesis conditions is essential to manipulate the morphological, structural, and magnetic properties of IONPs.^4,11^ Various synthesis routes, including physical, chemical, and biological methods, have been explored, with chemical methods like coprecipitation, hydrothermal, solvothermal, thermal decomposition, sol–gel, sonochemical decomposition, microemulsion, microwave-assisted, and electrochemical synthesis being extensively studied.^17,18^ Among these, hydrothermal synthesis is particularly notable for its scalability, reproducibility, low cost, and ability to produce homogeneous IONPs with low size dispersion and high crystallinity.^19,20^ However, despite efforts to fabricate magnetite nanoparticles with desired magnetic properties via hydrothermal methods, achieving precise control over anisotropic morphologies remains challenging, often resulting in spherical nanostructures with sizes ranging from 15 to 120 nm.^21^ Moreover, this method is limited in its ability to produce pure iron, preventing the attainment of high magnetization values. On the other hand, it is well-known that the final hematite particle morphology can be easily tuned by adjusting the concentrations of the reagents, temperature of the reaction, and addition of surfactants.^20,22^ Several authors have reported the successful fabrication of hematite nanoparticles with different shapes and sizes, such as cubes,^23^ spindle-like,^24^ rods,^25^ rings,^26^ platelets,^27^ ellipsoids,^28^ and rhombohedra,^27^ for instance, making them good candidates as templates for subsequent chemical reactions.

The shape and size of hematite nanoparticles are influenced by precursor type, concentration, reaction temperature, and surfactants. Hematite formation often involves phase transitions from akaganeite (β-FeOOH) or goethite (α-FeOOH), with FeCl_3_ yielding cubic particles of higher crystallinity, while Fe­(NO_3_)_3_ results in irregular shapes.^22,23,29,30^ Factors like phosphate and sulfate ions promote anisotropic growth by altering crystallographic axis growth rates, leading to diverse morphologies such as nanorods, nanodiscs, and nanotubes.^15,24,28^ Hydrothermal synthesis parameters, including ion concentrations and additives like ammonium-based compounds,^30,31^ oleic acid,^27^ urea,^20^ and sodium hydroxide,^25^ further refine nanoparticle size and crystallinity by controlling growth directions and dissolution processes.

Temperature and reaction time are critical factors influencing the hematite nanoparticle size and morphology. Studies show that higher temperatures generally increase particle size, with sizes ranging from 35 nm at 140 °C to 45 nm at 200 °C during hydrothermal synthesis.^32^ Elevated temperatures can also transition phases (e.g., α-FeOOH to α-Fe_2_O_3_) and promote the formation of uniform, hexagonal platelet-like structures at 230 °C.^33^ Additionally, amino acids as controlling agents influence particle size at lower temperatures (150 °C), with their effect diminishing at higher temperatures (200 °C).^19^ Small size variations are observed at intermediate temperatures (120–180 °C).^31^ These studies highlight that lower temperatures reduce nanoparticles’ crystallinity and may result in mixed phases. However, the underlying mechanisms have not been completely explored, as most research focuses on precursor and additive concentrations while fixing reaction temperatures. Further systematic investigations are needed to address this gap.

Given these considerations, a viable alternative to produce nanoparticles with high-magnetization features and controlled anisotropic shapes is the direct reduction of prefabricated hematite nanoparticles.^34^ However, all of the reported studies highlight a transition phase from hematite to magnetite, with no evidence of metallic iron formation. This underscores the importance of further investigating reduction parameters to yield the fabrication of iron-based nanoparticles starting from anisotropically shaped hematite produced by hydrothermal synthesis.

The structural and magnetic properties of the reduced nanoparticles are strictly dependent on the gas composition and partial pressure, reaction temperature, and intrinsic properties of the native ore.^35–38^ Within this framework, several works have investigated the role of hydrogen reduction in the context of the iron industry aimed at reducing the amount of CO_2_ pollution, revealing that the direct reduction of hematite, in its bulk form, with hydrogen, is a multistep process in which kinetics are strictly dependent on the temperature. At temperatures below 570 °C, it is assumed that the reduction of hematite progresses through an initial transformation into magnetite, followed by its final conversion to iron.^35,39–42^ An early study developed by Sastri and his group investigated the reduction of hematite ores at temperatures ranging from 300 to 500 °C, concluding that the reduction to metallic iron proceeded by a two-step mechanism via magnetite formation.^40^ Pineau et al. investigated the reduction of hematite pellets in the temperature range of 220–680 °C.^39^ Li et al., for example, investigated the chemical reduction of natural magnetite at temperatures ranging from 495 to 570 °C, revealing a single-step conversion to metallic iron. They found that the degree of reduction and reaction rate increased as temperature increased.^43^ The study developed by Du et al. aims at investigating the reduction kinetics of hematite ores at temperatures between 400 and 500 °C, revealing an increase in reduction rate with increasing temperature.^44^ Jozwiak and his group showed that complete reduction of hematite to the metallic iron phase could be achieved at temperatures as low as 380 °C in pure hydrogen.^41^ The study conducted by Hessels, investigating hematite reduction between 400 and 900 °C, revealed that full conversion to metallic iron was faster at 500 °C than at higher temperatures.^45^ Moreover, depending on the nature of the initial hematite ore, porous metallic iron can be formed as a product, being a characteristic of reduction in hydrogen performed at relatively low temperatures.^39,43,46,47^ Although reduction with hydrogen has been widely reported in the literature, few studies on the reduction of nanoparticles have been performed, revealing that at temperatures below 570 °C the reduction of hematite nanoparticles follows the behavior of bulk magnetite.^48^ However, depending on the composition and specific reduction conditions, different mechanisms were underlined, making straightforward comparison difficult. The high number and complexity of variables influencing the final product make it difficult to achieve a complete understanding of the process.^42^

In this study, we synthesized iron-based nanoparticles through a multistep process involving a first step of hydrothermal synthesis to obtain hematite nanoparticles with controlled shape morphologies, followed by a second step consisting of hydrogen-based direct reduction (HyDR) of the previously synthesized nanoparticles. HyDR usually consists of chemical reactions in a reductive atmosphere at a high temperature. This approach enabled the development of anisotropic nanoparticles with high saturation magnetizations. Several sizes and shapes of nanocubes, nanoellipses, and nanoneedles were obtained by controlling the hydrolysis of FeCl_3_ by optimizing NH_4_H_2_PO_4_ concentration and hydrothermal synthesis periods of 0.5 h. This study thus sets an original route toward the fabrication of highly crystallized anisotropic hematite nanoparticles within a remarkably short time frame. Furthermore, we demonstrated that HyDR performed at 480 °C produces porous nanoparticles with enhanced magnetic moments.

## 2 Materials and Methods

The iron-based synthesis method involved a two-step process: first, the fabrication of hematite (α-Fe_2_O_3_) nanoparticles via hydrothermal synthesis, followed by a thermal treatment in a reductive environment to produce Fe@Fe_3_O_4_ nanoparticles.

### 2.1 Step 1: Hydrothermal Synthesis of Hematite (α-Fe_2_O_3_) Nanoparticles

Hematite nanoparticles were fabricated by hydrothermal synthesis of iron­(III) chloride (FeCl_3_) in the presence of ammonium dihydrogen phosphate (NH_4_H_2_PO_4_). To produce nanoparticles of different shapes and sizes, specific amounts of NH_4_H_2_PO_4_ were added to a water-based solution of FeCl_3_ and stirred vigorously for 15 min. The ratios of the reagents in the solutions are listed in Table 1. Each solution was then transferred to a separate Teflon-lined stainless-steel autoclave of 45 mL. The autoclaves, filled up to 80% of their volume, were sealed and heated to 220 °C, with a ramp of 10 °C/min, for 30 min, promoting the formation of hematite nanoparticles. After the reaction, the autoclaves were allowed to cool to room temperature naturally. The resulting hematite nanoparticles were collected by centrifugation and washed several times with deionized water to remove any residual reactants. The nanoparticles were then dried at 60 °C in an oven and collected for further treatment.

**Table 1:** Parameters of Dissolutions

Samples (Step 1)	NH_4_H_2_PO_4_ (mmol)	FeCl_3_ (mmol)	NH_4_H_2_PO_4_/FeCl_3_	Samples (Step 2)
${\mathrm{NC}}_{\alpha -{\mathrm{Fe}}_{2}O_{3}}$	0	2.404	0	${\mathrm{NC}}_{\mathrm{Fe}@{\mathrm{Fe}}_{3}O_{4}}$
${\mathrm{NE}}_{\alpha -{\mathrm{Fe}}_{2}O_{3}}$	0.0182	2.404	0.007	${\mathrm{NE}}_{\mathrm{Fe}@{\mathrm{Fe}}_{3}O_{4}}$
${\mathrm{NN}}_{\alpha -{\mathrm{Fe}}_{2}O_{3}}$	0.0240	2.404	0.01	${\mathrm{NN}}_{\mathrm{Fe}@{\mathrm{Fe}}_{3}O_{4}}$

### 2.2 Step 2: Hydrogen-Based Direct Reduction (HyDR) in Controlled Atmosphere

The as-prepared samples from the three compositions synthesized via the hydrothermal method were subjected to HyDR^39,40^ of iron oxides (in this case hematite). The samples corresponding to the three different compositions were subsequently named ${\mathrm{NC}}_{\mathrm{Fe}@{\mathrm{Fe}}_{3}O_{4}}$ ${\mathrm{NE}}_{\mathrm{Fe}@{\mathrm{Fe}}_{3}O_{4}}$ , and ${\mathrm{NN}}_{\mathrm{Fe}@{\mathrm{Fe}}_{3}O_{4}}$ , respectively, as presented in Table 1. In this step, the dried and prepared hydrothermal nanoparticles were placed in a ceramic boat inside a tube furnace. The furnace was purged with nitrogen gas to remove any residual oxygen, and the temperature of the furnace was gradually increased to 480 °C. A gas mixture of 40% hydrogen and 60% argon was introduced into the furnace to create a reductive environment. The nanoparticles were subjected to this thermal treatment for 5 h, where the hydrogen gas served as the reducing agent, converting hematite (α-Fe_2_O_3_) into a mixture of iron (Fe) and magnetite (Fe_3_O_4_). After the reduction process, the furnace was allowed to cool to room temperature under an argon atmosphere to prevent reoxidation of the nanoparticles. The resulting iron–iron oxide nanoparticles were collected and stored in an inert atmosphere or vacuum to avoid reoxidation.

### 2.3 Characterization

The synthesized iron-based nanoparticles were characterized by using various techniques to confirm their composition, size, morphology, properties, and biocompatibility. Scanning electron microscopy (SEM, Phillips-FEI/Quanta 400 FEG high resolution) was used to examine the surface morphology and particle distribution, while X-ray diffraction (XRD, Rigaku-SmartLab-CBO-BB-FDS05deg) analysis was conducted to identify the crystal structure and phase composition within a 2θ range of 20°–80°. Rietveld refinements revealed phase quantities, lattice parameter values (*a,c*) and crystallite sizes (*D*_XRD_) for all the samples. Brunauer–Emmett–Teller (BET) was employed to determine the specific surface area and porosity through nitrogen adsorption isotherm measurements at 77 K. Mössbauer spectra of the nanoparticles were collected at room temperature in transmission mode using a conventional constant acceleration spectrometer and a 25-mCi^57^ Co source in Rh matrix to identify the specific iron oxide phases. The velocity scale was calibrated using α-Fe foil at room temperature. Isomer shift values, IS, are given relative to this standard. To further analyze the surface chemical composition and oxidation states, X-ray photoelectron spectroscopy (XPS, Phoibos 150 EP MCD analyzer, SPECS) was used. The samples were exposed to monochromatic Al Kα X-rays with an energy of 1486.6 eV. The emission angle relative to the sample surface was maintained at 90°. Pass energies of 50 and 20 eV were employed for recording the survey spectrum and the core-level spectrum, respectively. Finally, superconducting quantum interference device (SQUID, MPMS3 quantum design) magnetometry was used to assess the magnetic properties of the nanoparticles. Magnetization versus applied magnetic field (*M*-*H*) measurements were carried out under the zero-field-condition at several temperatures, as discussed in the next session. In each hysteresis loop cycle, the magnetization data was recorded while the applied field varied between −5 and 5 T. In the zero-field cooling (ZFC) protocol, the temperature of the sample was cooled from room temperature to a particular temperature in the absence of any applied magnetic field, while in the field cooling (FC), the sample was cooled in the presence of a small magnetic field (100 Oe). After that, a fixed magnetic field (100 Oe) was applied, and the magnetic moment was measured as a function of temperature. To assess the biocompatibility of the fabricated nanoparticles for biomedical applications, the U-87 MG cell line was selected. The cells were placed in a 96-well microplate at 40,000 cells/well in 100 μL supplemented DMEM and incubated at 37 °C in a 5% CO_2_ atmosphere with different concentrations of nanoparticles, from 2.5 to 100 μg/mL. To evaluate cell viability, an Alamar Blue assay was performed at two different time points: 48 and 72 h post-incubation.

## 3 Results and Discussion

### 3.1 Morphological Characterization

#### 3.1.1 Scanning Electron Microscopy (SEM)

SEM images of the samples synthesized with different amounts of NH_4_H_2_PO_4_ (0, 0.018, and 0.024 mmol) after the first step, which corresponds to the hydrothermal synthesis (as-prepared α-Fe_2_O_3_), as presented in Figure 1, reveal the formation of monodisperse nanoparticles with distinct morphologies. Specifically, nanocubes (sample ${\mathrm{NC}}_{\alpha -{\mathrm{Fe}}_{2}O_{3}}$ , Figure 1a), nanoellipses (sample ${\mathrm{NE}}_{\alpha -{\mathrm{Fe}}_{2}O_{3}}$ , Figure 1b), and nanoneedles (sample ${\mathrm{NN}}_{\alpha -{\mathrm{Fe}}_{2}O_{3}}$ , Figure 1c) were observed for NH_4_H_2_PO_4_ concentrations of 0, 0.018, and 0.024 mmol, respectively. The addition of NH_4_H_2_PO_4_ significantly influences the morphological evolution of hydrothermal nanoparticles, transitioning from cubic nanoparticles to increasingly elongated morphologies. This progression is characterized by a shift from nanocubes with an average size of 360 ± 23 nm to nanoellipses with a major axis length of 1190 ± 73 nm and ultimately to nanoneedles, with an average length of 1030 ± 61 nm and a width of 440 ± 34 nm, with increasing NH_4_H_2_PO_4_ concentration. From this analysis, it can be concluded that with increasing amounts of NH_4_H_2_PO_4_, an increase in the length-to-diameter ratio is also observed, being 1, 1.75, and 2.33 for nanocubes (NC), nanoellipses (NE), and nanoneedles (NN), respectively.

**Figure 1:**
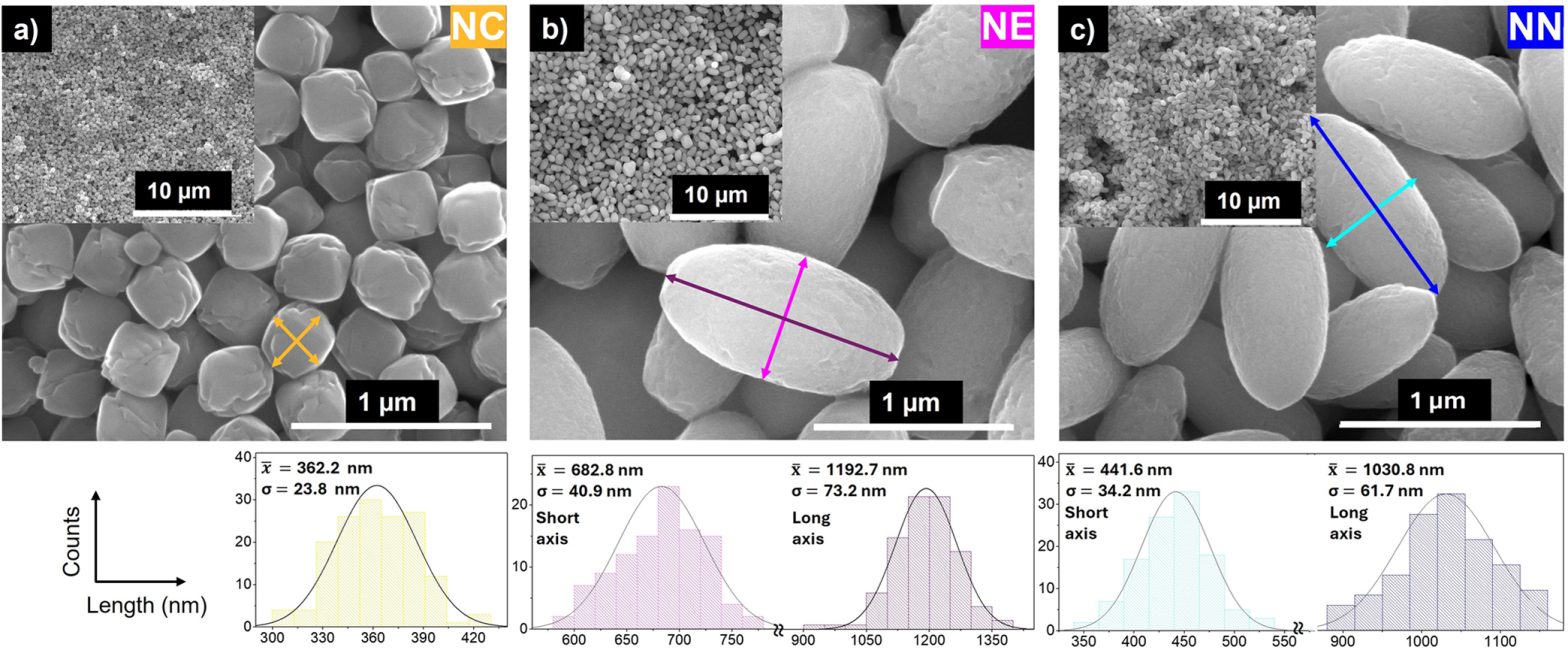
Hematite nanoparticles (α-Fe_2_O_3_) prepared in the first step with different amounts of NH_4_H_2_PO_4_: (a) nanocubes (NC), (b) nanoellipses (NE), and (c) nanoneedles (NN) and respective size distributions. *x* = average; σ = STD; SE = standard error.

From Figure 2, it can be noticed that the nanoparticles after the HyDR performed in step 2 of the synthesis process are characterized by a reduction in size (histograms in Figure 2), invariant morphology, and apparent enhancement of porosity. The overall size reduction of the nanoparticles obtained after the HyDR can be attributed to the kinetics of the process. Kinetic studies have shown that, at temperatures below 570 °C, the reduction of hematite proceeds toward its initial transformation to magnetite. This step is followed by a final conversion to iron,^35,39–42^ being the overall rate of the process generally controlled by the topochemical reduction of magnetite.^40^ However, if the particles are small enough, the rate-determining step was found to change from phase boundary to nucleation, which is associated with a uniform internal reduction.^48,49^ Therefore, during the reduction of α-Fe_2_O_3_ with hydrogen at 480 °C in the second step of the process, it is likely that the total amount (or majority) of α-Fe_2_O_3_ was rapidly converted to Fe_3_O_4_ through an exothermic reaction. Then, the direct reduction of magnetite to porous metallic iron (characteristic of hydrogen-induced reduction at relatively low temperatures)^39,43,46^ should occur more slowly, governed by a phase-boundary reaction which was carried out over an extended annealing period (5 h) under the reducing atmosphere, resulting in the nanoparticles’ size reduction due to oxygen loss. The difference in the reduction rate of the two iron oxides is attributed to the intrinsic nature of magnetite, which possesses a hard and dense shell that slows down the diffusion of the inlet gas into the bulk structure.^36^ Within this framework, it can be stated that HyDR of iron oxides is a gradual multistep process that depends on several factors, such as temperature, pressure, gas composition, and particle size, for instance.^38,41^

**Figure 2:**
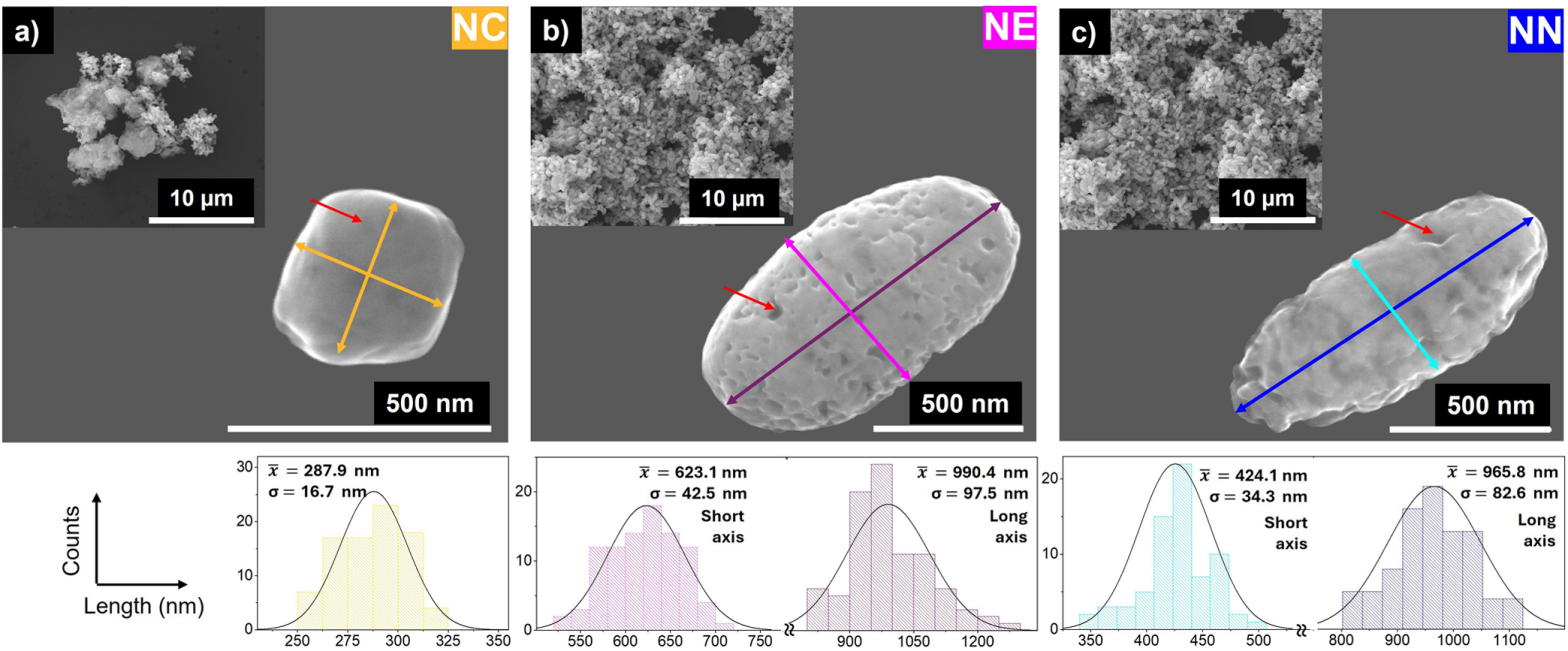
Iron-based nanoparticles (Fe@Fe_3_O_4_) prepared in step 2 after the HyDR: (a) nanocubes (NC), (b) nanoellipses (NE), and (c) nanoneedles (NN) and respective size distributions. *x* = average; σ = STD; SE = standard error. Red arrows exemplify the pores generated after the reduction step.

#### 3.1.2 Porosity

The Brunauer–Emmett–Teller (BET) technique was employed to determine the specific surface area through nitrogen adsorption isotherm measurements conducted at 77 K (Figure S1). Assuming multilayer gas adsorption on the adsorbent’s surface,^50^ the key parameters obtained from this analysis are shown in Figure 3. It compares the micropore area and the external surface area (together forming the total BET surface area), as well as the micropore volume and pore diameter, as a function of the type of nanostructure before (hematite) and after the reduction process (iron-based).

**Figure 3:**
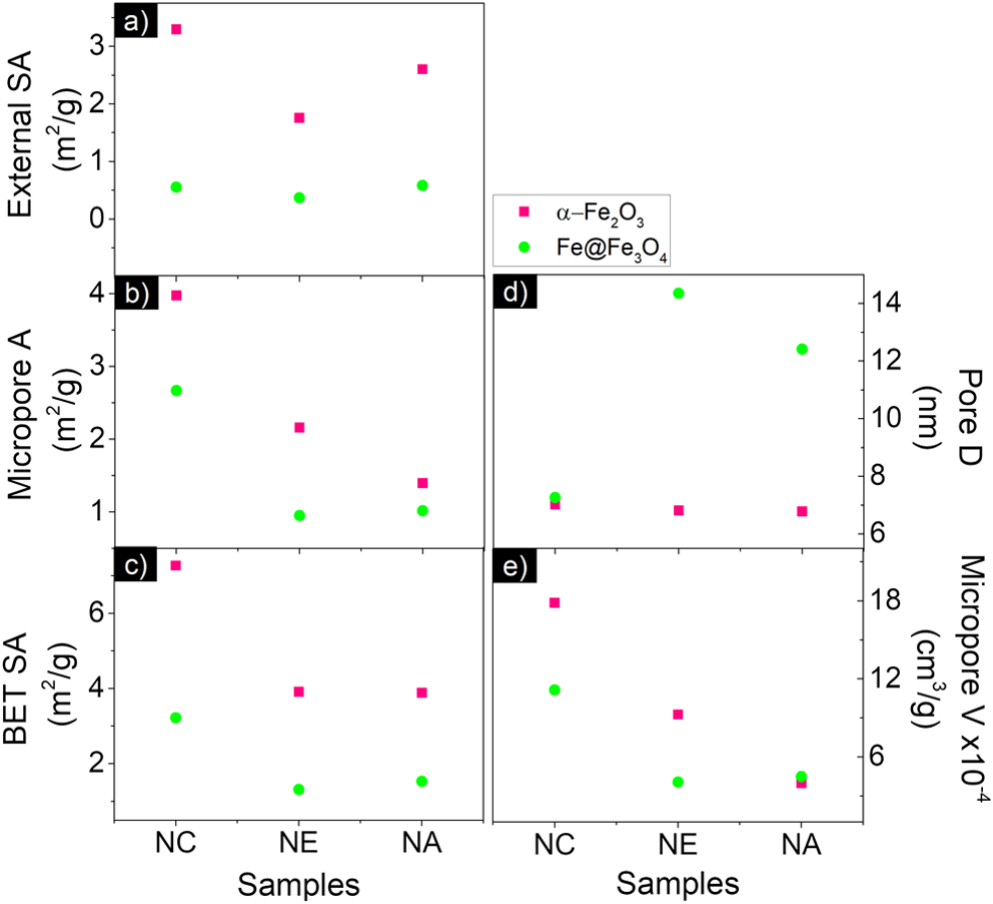
Compared analysis of all samples (α-Fe_2_O_3_ and Fe@Fe_3_O_4_) by BET providing data on (a) external surface area (External SA), (b) micropore area (*A*), (c) BET surface area (BET SA), (d) Pore diameter (*D*), (e) micropore volume (*V*). BET SA = External SA + A.

The analysis shows a significant reduction in both the micropore area and the external surface area following chemical reduction. At first glance, this may seem contradictory, especially since SEM images visibly indicate that all nanoparticles acquire additional porosity after the reduction process. However, detailed insights from BET analysis of hematite samples reveal that these structures inherently possess substantial porosity, with most pore sizes measuring less than 7 nm. Such small-scale features are challenging to detect with standard microscopy, leading to potential misinterpretations when relying solely on visual observations.

The underlying explanation lies in the inherent characteristics of the materials presented in each fabrication step. The hematite nanoparticles formed through the hydrothermal method naturally exhibit a porous structure. However, after the gaseous reduction process, the BET surface area often decreases due to sintering effects. This effect results from the collapse of native pores as oxygen is removed, and phase transformations occur that do not conserve volume. These processes compact the structure, diminishing the overall surface area while increasing the density of the material.^38,48,51^ Furthermore, the characteristics and behavior of the pores formed during reduction are highly dependent on the reduction temperature and the partial pressure of hydrogen used in the process.^42,46,47^ Low-temperature reductions, typically below 570 °C, produce a distinct product morphology characterized by fine and elongated pores. This specific pore structure is indicative of thermal reduction conditions where wüstite (FeO) is thermodynamically unstable.^37^ Such morphological traits highlight the complex balance among temperature, chemical reactivity, and physical transformations during reduction. The collapsing of native pores coupled with phase transitions can result in surface area reduction despite the overall appearance of increased porosity observed via SEM. Early works conducted by Turkdogan and his group reported that pore surface area decreases with increasing reduction temperature.^47^ The same behavior was observed by Fortini et al., which also postulated that in the absence of sintering, the total pore volume would have been subjected to an increase of 2.23%, so that a decrease in porous surface area is an indication of sintering.^39,51^ This intricate interaction between sintering effects and phase changes underscores the importance of using complementary analysis techniques, such as BET and SEM, to achieve a comprehensive understanding of the nanostructure porosity before and after chemical reduction.

### 3.2 Structural Characterization

This section summarizes the crystallographic, surface, and nuclear structure of the fabricated sample.

#### 3.2.1 Crystallographic Characterization

Figure 4 shows the XRD diffractogram patterns, along with Rietveld refinement for the as-prepared samples ${\mathrm{NC}}_{\alpha -{\mathrm{Fe}}_{2}O_{3}}$ , ${\mathrm{NE}}_{\alpha -{\mathrm{Fe}}_{2}O_{3}}$ , and ${\mathrm{NN}}_{\alpha -{\mathrm{Fe}}_{2}O_{3}}$ , obtained after the hydrothermal method in the first step, and Figure 5, after the second step, HyDR method, for iron-based NPs— ${\mathrm{NC}}_{\mathrm{Fe}@{\mathrm{Fe}}_{3}O_{4}}$ , ${\mathrm{NE}}_{\mathrm{Fe}@{\mathrm{Fe}}_{3}O_{4}}$ , and ${\mathrm{NN}}_{\mathrm{Fe}@{\mathrm{Fe}}_{3}O_{4}}$ . After the hydrothermal synthesis, all three samples show the hematite crystallographic phase (Figure 4) which corresponds to a rhombohedral crystal structure (space group *R*3̅*c*),^52^ consistent with Crystallography Open Database (COD) card No. 9015964 for hematite. For all these samples— ${\mathrm{NC}}_{\alpha -{\mathrm{Fe}}_{2}O_{3}}$ , ${\mathrm{NE}}_{\alpha -{\mathrm{Fe}}_{2}O_{3}}$ , ${\mathrm{NN}}_{\alpha -{\mathrm{Fe}}_{2}O_{3}}$ —strong diffraction peaks were identified at 2θ = 24.1°, 33.1°, 35.6°, 40.8°, 49.4°, 54.0°, 57.5°, 62.4°, and 64.0°, corresponding to Bragg reflections (012), (104), (110), (113), (024), (116), (018), (214), and (300), respectively, as usually reported.^53^

**Figure 4:**
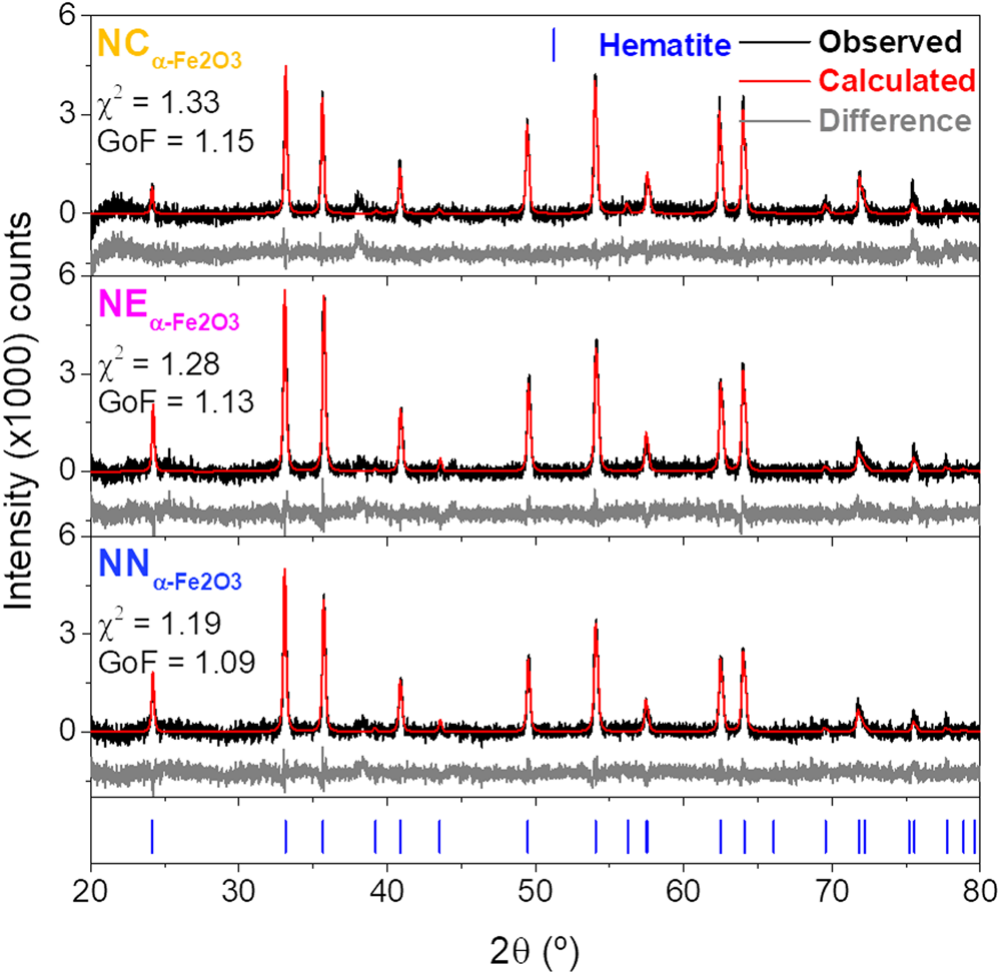
X-ray diffraction patterns of the hematite nanoparticles prepared with different amounts of NH_4_H_2_PO_4_— ${\mathrm{NC}}_{\alpha -{\mathrm{Fe}}_{2}O_{3}}$ , ${\mathrm{NE}}_{\alpha -{\mathrm{Fe}}_{2}O_{3}}$ , and ${\mathrm{NN}}_{\alpha -{\mathrm{Fe}}_{2}O_{3}}$ . The experimental diffractograms (solid black line), Rietveld refinements fitting results (solid red line), and difference (gray solid line) are represented, and the corresponding χ^2^ and goodness-of-fit indicator (GoF = *R*_wp_/*R*_exp_) are shown.

**Figure 5:**
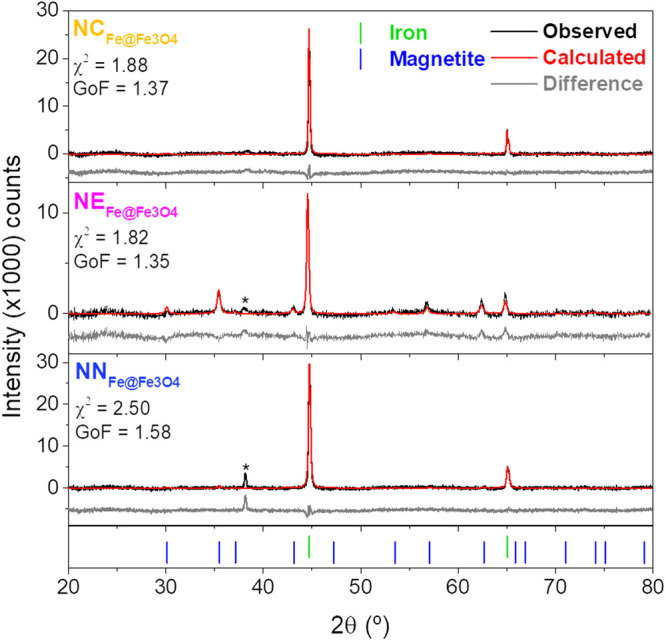
X-ray diffraction patterns of the iron-based nanoparticles prepared in step 2 after the HyDR— ${\mathrm{NC}}_{\mathrm{Fe}@{\mathrm{Fe}}_{3}O_{4}}$ , ${\mathrm{NE}}_{\mathrm{Fe}@{\mathrm{Fe}}_{3}O_{4}}$ , and ${\mathrm{NN}}_{\mathrm{Fe}@{\mathrm{Fe}}_{3}O_{4}}$ . The experimental diffractograms (solid black line), Rietveld refinements fitting results (solid red line), and difference (gray solid line) are represented, and the corresponding χ^2^ and goodness-of-fit indicator (GoF = *R*_wp_/*R*_exp_) are shown. (*) represents the peak corresponding to the substrate.

These peaks confirm the high crystallinity of the synthesized nanoparticles for all shape morphologies, including nanocubes, nanoellipses, and nanoneedles. However, it can be noticed that while the preferential crystallographic direction of samples ${\mathrm{NC}}_{\alpha -{\mathrm{Fe}}_{2}O_{3}}$ and ${\mathrm{NN}}_{\alpha -{\mathrm{Fe}}_{2}O_{3}}$ is the (104), whose peak appears more intense when compared to the (110), in sample ${\mathrm{NE}}_{\alpha -{\mathrm{Fe}}_{2}O_{3}}$ an increase in intensity of the (110) direction appears.

Furthermore, the peak broadening is dominated by crystallite particle size (*D*_XRD_) in the 60–52 nm range, with no significant influence from the lattice microstrain (Table 2). This is a typical occurrence when the NPs are synthesized by forced hydrolysis.^53^ The lattice parameters *a* and *c* show typical dimensions in accordance with the literature for the hematite crystallographic phase.^52,53^

**Table 2:** Rietveld Refinement Results of the Hematite Nanoparticles Prepared in the First Step with Different Amounts of NH_4_H_2_PO_4_— ${\mathrm{NC}}_{\alpha -{\mathrm{Fe}}_{2}O_{3}}$ , ${\mathrm{NE}}_{\alpha -{\mathrm{Fe}}_{2}O_{3}}$ , and ${\mathrm{NN}}_{\alpha -{\mathrm{Fe}}_{2}O_{3}}$

	Hematite				
Sample	*a* (Å)	*c* (Å)	*D*_XRD_ (nm)	ε (%)	Ratio (104)/(110)
${\mathrm{NC}}_{\alpha -{\mathrm{Fe}}_{2}O_{3}}$	5.038 ± 0.029	13.769 ± 0.079	60.27 ± 0.68	0	1.24
${\mathrm{NE}}_{\alpha -{\mathrm{Fe}}_{2}O_{3}}$	5.040 ± 0.021	13.794 ± 0.058	52.51 ± 0.50	0	1.00
${\mathrm{NN}}_{\alpha -{\mathrm{Fe}}_{2}O_{3}}$	5.039 ± 0.022	13.799 ± 0.059	53.04 ± 0.56	0	1.20

Figure 5 shows the XRD diffractogram patterns, along with Rietveld refinement for the samples ${\mathrm{NC}}_{\mathrm{Fe}@{\mathrm{Fe}}_{3}O_{4}}$ , ${\mathrm{NE}}_{\mathrm{Fe}@{\mathrm{Fe}}_{3}O_{4}}$ , ${\mathrm{NN}}_{\mathrm{Fe}@{\mathrm{Fe}}_{3}O_{4}}$ , after the reduction step ( ${\mathrm{NE}}_{\mathrm{Fe}@{\mathrm{Fe}}_{3}O_{4}}$ and ${\mathrm{NN}}_{\mathrm{Fe}@{\mathrm{Fe}}_{3}O_{4}}$ show a peak around 38.1°, which is indexed to the sample holder; therefore, we omitted that peak for the refinement). All the reduced samples show the cubic spinel structure of magnetite (space group *Fd3̅m*, COD card No. 9002673)^52^ and the body-centered cubic (BCC) structure of metallic iron (space group *Im3̅m*, COD card No. 9008536). Peaks corresponding to magnetite were observed at 30.1° (220), 35.5° (311), 43.1° (400), 57.0° (511), and 62.6° (440),^54^ while iron peaks were detected at 44.7° (110) and 65.1° (200).^55^ Rietveld refinements confirmed the phase composition, with nanoneedles $\left({\mathrm{NN}}_{\mathrm{Fe}@{\mathrm{Fe}}_{3}O_{4}}\right)$ showing the highest iron content (97.3%), followed by nanocubes ( ${\mathrm{NC}}_{\mathrm{Fe}@{\mathrm{Fe}}_{3}O_{4}}$ , 96.7%) and nanoellipses ( ${\mathrm{NE}}_{\mathrm{Fe}@{\mathrm{Fe}}_{3}O_{4}}$ , 62.3%), see Table 3. Additionally, lattice parameters of all magnetite samples are in conformity with the literature,^52^ as well as the lattice parameter of the iron phase.^55^

**Table 3:** Rietveld Refinement Results of the Iron-Based Nanoparticles Prepared in Step 2 after the HyDR— ${\mathrm{NC}}_{\mathrm{Fe}@{\mathrm{Fe}}_{3}O_{4}}$ , ${\mathrm{NE}}_{\mathrm{Fe}@{\mathrm{Fe}}_{3}O_{4}}$ , and ${\mathrm{NN}}_{\mathrm{Fe}@{\mathrm{Fe}}_{3}O_{4}}$

	Magnetite				Iron			
Sample	Phase Quantity (%)	*a* (Å)	*D*_XRD_ (nm)	ε (%)	Phase Quantity (%)	*a* (Å)	*D*_XRD_ (nm)	ε (%)
${\mathrm{NC}}_{\mathrm{Fe}@{\mathrm{Fe}}_{3}O_{4}}$	3.30 ± 0.84	8.40 ± 0.36	-	0	96.70 ± 0.84	2.87 ± 0.12	84.8 ± 1.2	0
${\mathrm{NE}}_{\mathrm{Fe}@{\mathrm{Fe}}_{3}O_{4}}$	37.68 ± 0.86	8.43 ± 0.33	25.4 ± 1.3	0	62.32 ± 0.86	2.88 ± 0.11	47.79 ± 0.80	0
${\mathrm{NN}}_{\mathrm{Fe}@{\mathrm{Fe}}_{3}O_{4}}$	2.74 ± 0.76	8.40 ± 0.48	-	0	97.26 ± 0.76	2.87 ± 0.16	54.28 ± 0.81	0

The crystallite sizes of hematite nanoparticles showed a dependence on NH_4_H_2_PO_4_ concentrations. The ${\mathrm{NC}}_{\alpha -{\mathrm{Fe}}_{2}O_{3}}$ exhibited the largest crystallite size (60.27 ± 0.68 nm), whereas the ${\mathrm{NE}}_{\alpha -{\mathrm{Fe}}_{2}O_{3}}$ and ${\mathrm{NN}}_{\alpha -{\mathrm{Fe}}_{2}O_{3}}$ had slightly smaller crystallite sizes, approximately 52–53 nm (Figure 6). This variation correlates inversely with changes in particle aspect ratio (particle length/width ratio) observed in SEM images (Figure 1) and plotted in Figure 6.

**Figure 6:**
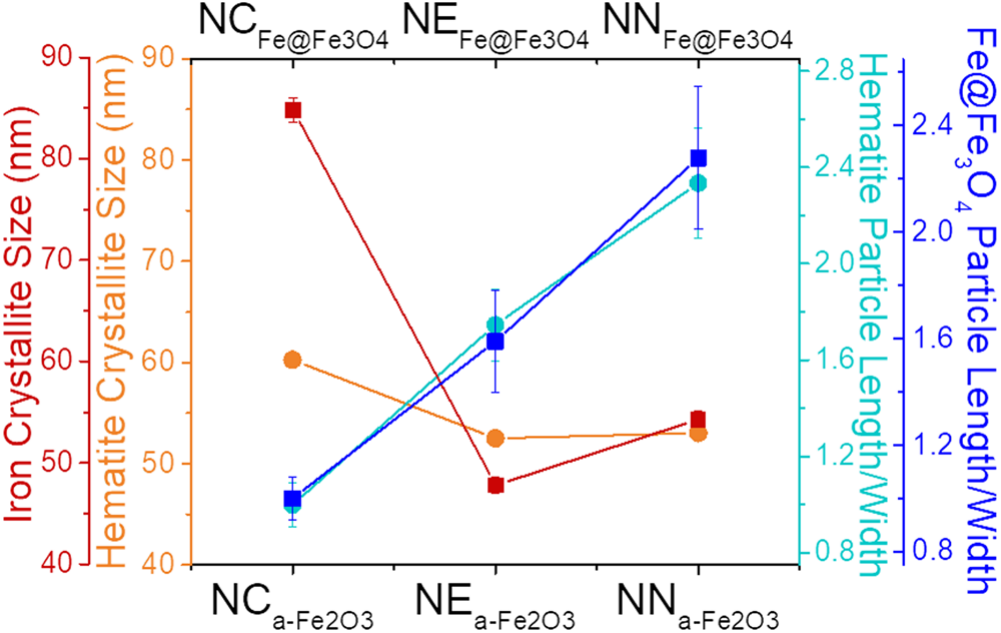
Hematite crystallite size (orange circles), iron crystallite size (red squares), hematite particle length/width ratio (light blue circles), and Fe@Fe_3_O_4_ particle length/width ratio (dark blue squares) for the nanoparticles fabricated in step 1 (α-Fe_2_O_3_) and reduced in step 2 by HyDR (Fe@Fe_3_O_4_) samples—nanocubes (NC), nanoellipses (NE), and nanoneedles (NN).

For the reduced samples, a similar behavior is observed, where the ${\mathrm{NC}}_{\mathrm{Fe}@{\mathrm{Fe}}_{3}O_{4}}$ sample has the smallest particle size and the largest iron crystallite size (84.8 ± 1.2 nm), whereas the ${\mathrm{NE}}_{\mathrm{Fe}@{\mathrm{Fe}}_{3}O_{4}}$ and ${\mathrm{NN}}_{\mathrm{Fe}@{\mathrm{Fe}}_{3}O_{4}}$ samples have similar particle sizes and iron crystallite sizes (47–54 nm), as shown in Figure 6. The iron crystallites have larger dimensions in the reduced samples than in the original hematite crystallites for the nanocubes sample ( ${\mathrm{NC}}_{\alpha -{\mathrm{Fe}}_{2}O_{3}}$ and ${\mathrm{NC}}_{\mathrm{Fe}@{\mathrm{Fe}}_{3}O_{4}}$ ), leading us to the conclusion that during the thermal treatment there is, in part, an aggregation of the crystallites. However, for the nanoellipses ( ${\mathrm{NE}}_{\alpha -{\mathrm{Fe}}_{2}O_{3}}$ and ${\mathrm{NE}}_{\mathrm{Fe}@{\mathrm{Fe}}_{3}O_{4}}$ ) and the nanoneedles ( ${\mathrm{NN}}_{\alpha -{\mathrm{Fe}}_{2}O_{3}}$ and ${\mathrm{NN}}_{\mathrm{Fe}@{\mathrm{Fe}}_{3}O_{4}}$ ) samples, the crystallite sizes of the original hematite and iron are identical. The smaller size of the nanocubes could facilitate energy transfer between adjacent crystallites, promoting faster aggregation. In contrast, the hematite nanoellipses and nanoneedles, with their elongated morphology and possibly higher thermal stability, require more thermal energy to overcome the barriers for aggregation.

#### 3.2.2 Surface Composition Analysis

From this point forward, we will focus exclusively on analyzing the iron-based samples fabricated in step 2 through HyDR. The surface, nuclear structure, and magnetic properties of the precursor samples fabricated in step 1 do not provide any useful information for the reduced samples and will no longer be considered.

To study the surface composition of the reduced samples, fabricated in step 2 after the HyDR, X-ray photoelectron spectroscopy (XPS) characterization was carried out. The spectral region corresponding to the Fe 2p photoemission peak is shown in Figure 7. All spectra were calibrated using the adventitious C 1s peak set at a fixed value of 284.8 eV. A Shirley-type background was applied to the Fe 2p survey spectra to minimize most of the extrinsic loss structure.^56^ For all the samples, the Fe 2p_3/2_ envelope was fitted using the four most prominent peaks from the Gupta and Sen (GS) multiplet for high-spin Fe^3+^ and supplemented with the main GS multiplet peaks of high-spin Fe^2+^, while the Fe 2p_1/2_ envelope included the three primary peaks for Fe^3+^ and the most intense one associated with Fe^2+^ of the GS multiplets.^57,58^ Additionally, shakeup satellites were considered for both spin–orbit components. For each component, the peak position, FWHM, distribution, and percentage area are reported in Table S2. Fittings have been performed assuming 40% of a Lorentzian distribution in the peak shape, while the asymmetric peak shape associated with Fe(0) was defined by a standard metal iron sample (LA­(1.2,4.8,3)).^59^ The surface concentration in the sample is much lower than that of Fe^3+^ and Fe^2+^ in all the reported samples. Moreover, the amount of metallic iron (Fe(0)) on the surface of the samples is much lower than the values reported in the XRD analysis. This discrepancy can be explained by the fact that XPS is a surface-sensitive technique, providing information limited to the outermost atomic layers of the nanoparticles. Consequently, the XPS results suggest that the surface of the nanoparticles is predominantly composed of iron oxides. However, the XRD analysis indicates a substantial presence of metallic Fe within the nanoparticles. Combining both results suggests the hypothesis of a core–shell structure, where the nanoparticles consist of a metallic Fe core surrounded by an oxide shell.

**Figure 7:**
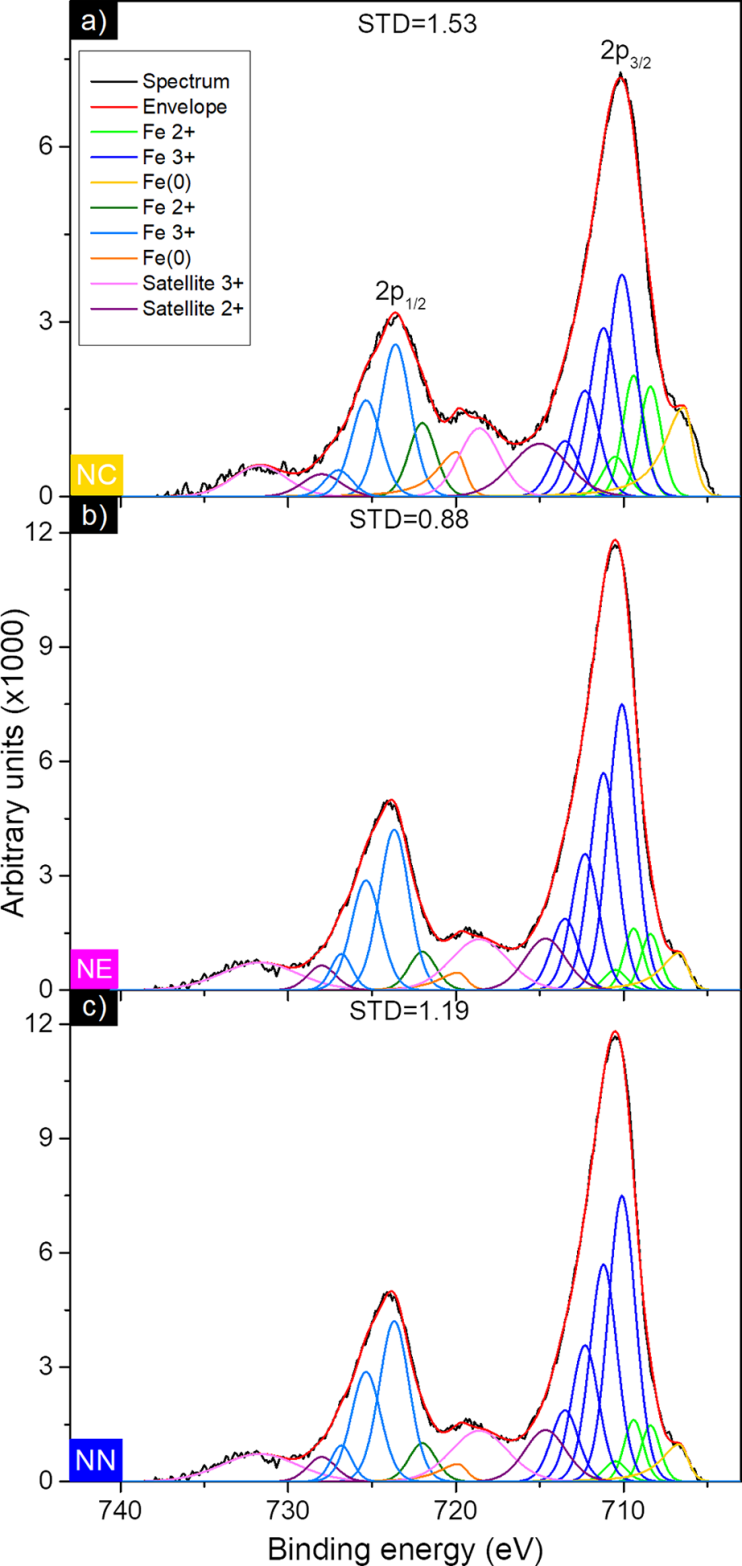
XPS 2p spectra of the iron-based Fe@Fe_3_O_4_ samples prepared in step 2 after the HyDR. (a) NC, (b) NE, and (c) NN.

#### 3.2.3 Nuclear Structure Analysis

To assess the specific chemical and structural properties of the bulk reduced samples, in particular, the determination of the Fe oxidation states, Mössbauer analysis was performed. The spectra of all samples, fabricated after HyDR in step 2, are presented in Figure 8 and were fitted to Lorentzian lines using a nonlinear least-squares method.^60^ Relative areas and line widths of both peaks in a quadrupole doublet and of peak pairs 1–6, 2–5, and 3–4 in a magnetic sextet were constrained to remain equal during the refinement procedure. After the reduction, all three samples presented magnetic sextets characteristics of metallic αFe^61^ (Table 4, green line in Figure 8), with ${\mathrm{NC}}_{\mathrm{Fe}@{\mathrm{Fe}}_{3}O_{4}}$ and ${\mathrm{NN}}_{\mathrm{Fe}@{\mathrm{Fe}}_{3}O_{4}}$ showing higher Fe component percentages (80% and 78%, respectively), consistent with the findings from XRD Rietveld analysis. Additionally, the presence of magnetite was confirmed for all samples by XRD (see Section 3.2.1) and was further indicated by the Mössbauer spectra for ${\mathrm{NE}}_{\mathrm{Fe}@{\mathrm{Fe}}_{3}O_{4}}$ and ${\mathrm{NN}}_{\mathrm{Fe}@{\mathrm{Fe}}_{3}O_{4}}$ (blue line in Figure 8b,c), as explained below. In the case of ${\mathrm{NC}}_{\mathrm{Fe}@{\mathrm{Fe}}_{3}O_{4}}$ , the amount of magnetite detected by XRD analysis was below the detection limit of Mössbauer spectroscopy (<2%) and the most intense, resolved peaks of this oxide, at approximately −8 mm/s and +8 mm/s, are within the range of the experimental error. However, we can anticipate that SQUID measurements evidence the Verwey transition of magnetite in the three ${\mathrm{NC}}_{\mathrm{Fe}@{\mathrm{Fe}}_{3}O_{4}}$ , ${\mathrm{NE}}_{\mathrm{Fe}@{\mathrm{Fe}}_{3}O_{4}}$ , and ${\mathrm{NN}}_{\mathrm{Fe}@{\mathrm{Fe}}_{3}O_{4}}$ samples (see Section 3.3).

**Figure 8:**
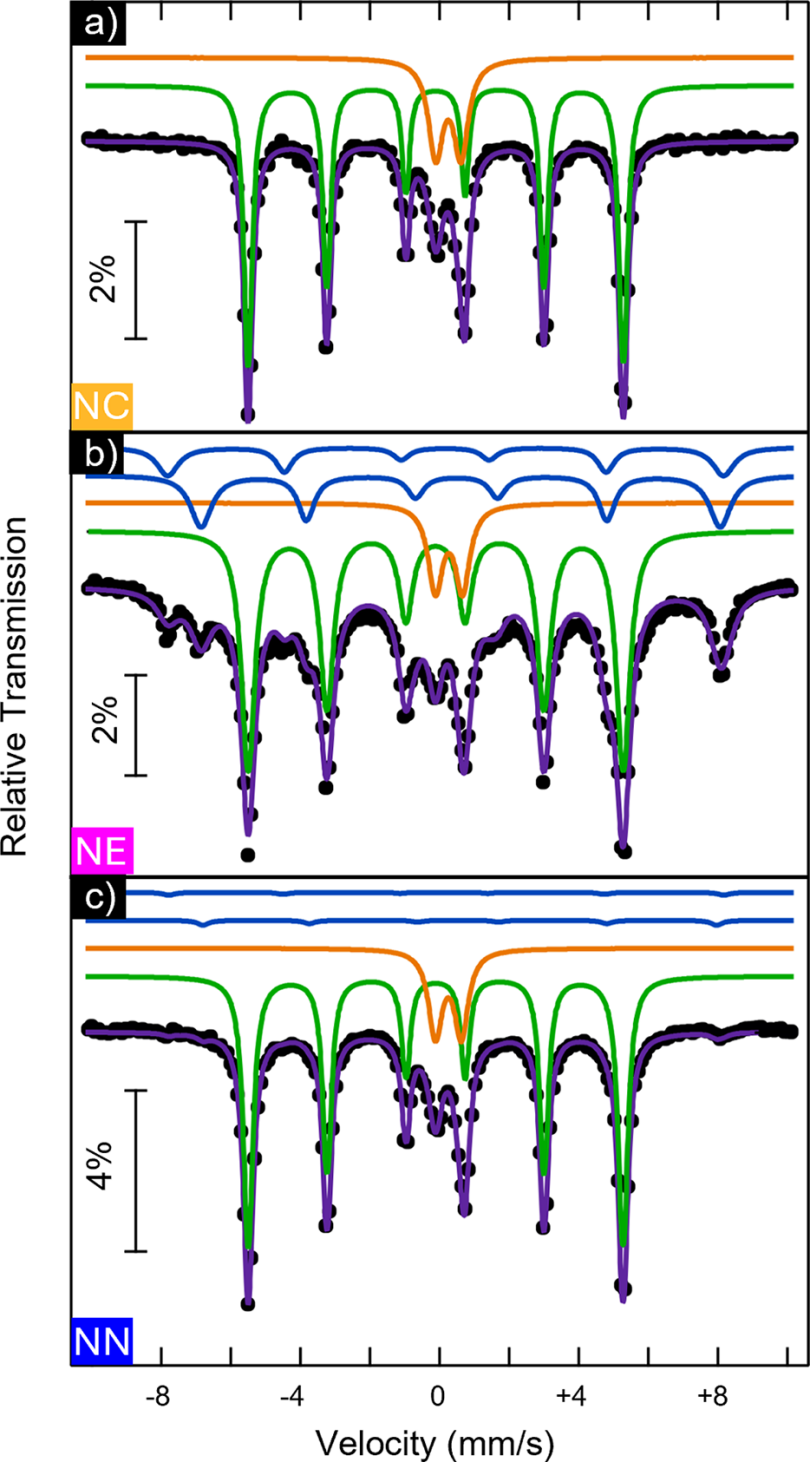
Mössbauer spectra of the iron-based Fe@Fe_3_O_4_ samples prepared in step 2 after the HyDR. (a) NC, (b) NE, and (c) NN. Green line—αFe sextet, blue line—Fe_3_O_4_ sextet, orange line—Fe^3+^ oxide doublet.

**Table 4:** Mössbauer Estimated Parameters of the Iron-Based Fe@Fe_3_O_4_ Samples Prepared in Step 2 after the HyDR

Sample	IS^a^ mm/s	QS^b^ ε, mm/s	*B*_hf_^c^ T	Γ^d^ mm/s	Fe species	*I* ^e^
${\mathrm{NC}}_{\mathrm{Fe}@{\mathrm{Fe}}_{3}O_{4}}$	0.00	0.00	33.4	0.25	metallic αFe	78%
	0.37	0.73	-	0.50	Fe^3+^ CN^f^ = 6 oxide	22%
${\mathrm{NE}}_{\mathrm{Fe}@{\mathrm{Fe}}_{3}O_{4}}$	0.00	0.01	33.4	0.47	metallic αFe	60%
	0.66	0.12	46.3	0.57	Fe^2.5+^ CN = 6 Fe_3_O_4_	19%
	0.28	0.01	49.6	0.57	Fe^3+^ CN = 4 Fe_3_O_4_	10%
	0.39	0.78	-	0.50	Fe^3+^ CN = 6 oxide	11%
${\mathrm{NN}}_{\mathrm{Fe}@{\mathrm{Fe}}_{3}O_{4}}$	0.00	0.01	33.4	0.29	metallic αFe	80%
	0.67	0.05	45.8	0.37	Fe^2.5+^ CN = 6 Fe_3_O_4_	1%
	0.27	0.07	49.5	0.37	Fe^3+^ CN = 4 Fe_3_O_4_	1%
	0.37	0.75	-	0.47	Fe^3+^ CN = 6 oxide	18%

a IS, isomer shifts relative to metallic α-Fe at 298 K.

b QS quadrupole splitting and ε = (*e* ^2^ *QV*_*zz*_/4) (3cos^2^θ – 1) quadrupole shift estimated for quadrupole doublets and magnetic sextets, respectively. Estimated errors ≤0.02 mm/s for IS, ε, Γ; <0.3 T for *B*_hf_; and <2% for *I*.

c *B*_hf_, magnetic hyperfine field.

d Γ, line width.

e *I*, relative area.

f CN, coordination number.

The spectrum of the ${\mathrm{NC}}_{\mathrm{Fe}@{\mathrm{Fe}}_{3}O_{4}}$ sample (Figure 8a) may be analyzed by one sextet and one doublet. The estimated parameters (Table 4) show that the sextet is due to metallic αFe^61^ and the doublet to a Fe^3+^ containing compound.^62^ Considering the composition of these samples, this compound is a Fe^3+^ oxide.^63,64^ The determined percentage of metallic αFe of 78% is in good agreement with the XRD data, where it was also found that a major part of the sample is composed of metallic Fe with a small percentage of iron oxide.

In addition to the above-referred sextet and doublet, the spectra of the ${\mathrm{NN}}_{\mathrm{Fe}@{\mathrm{Fe}}_{3}O_{4}}$ and ${\mathrm{NE}}_{\mathrm{Fe}@{\mathrm{Fe}}_{3}O_{4}}$ samples evidence two more sextets (blue line, Figure 8b,c). The deduced parameters (Table 4) are typical of Fe^2.5+^ in octahedral coordination and Fe^3+^ in tetrahedral coordination in magnetite.^63,64^ The relative Fe fractions contributing to these two sextets are consistent with nonoxidized magnetite with the ideal stoichiometry.

### 3.3 Magnetic Characterization

Magnetic measurements of the reduced samples fabricated in step 2 were performed as a function of either temperature or applied field.

#### 3.3.1 Magnetization as a Function of the Applied Magnetic Field

The M-H hysteresis loops at *T* = 300 and 5 K, for samples ${\mathrm{NC}}_{\mathrm{Fe}@{\mathrm{Fe}}_{3}O_{4}}$ , ${\mathrm{NE}}_{\mathrm{Fe}@{\mathrm{Fe}}_{3}O_{4}}$ , and ${\mathrm{NN}}_{\mathrm{Fe}@{\mathrm{Fe}}_{3}O_{4}}$ are shown in Figure 9a,b, respectively. The hysteresis loops for all of the samples show the ferromagnetic nature of the ferrite-based magnetic nanoparticles. As expected for ferromagnetic multidomain nanoparticles,^9^ a decrease in temperature results in an increase in saturation magnetization, coercivity, and remanent magnetization, as recorded in Table 5 for M-H loops at 5 and 300 K and presented in detail in Figure 10.

**Figure 9:**
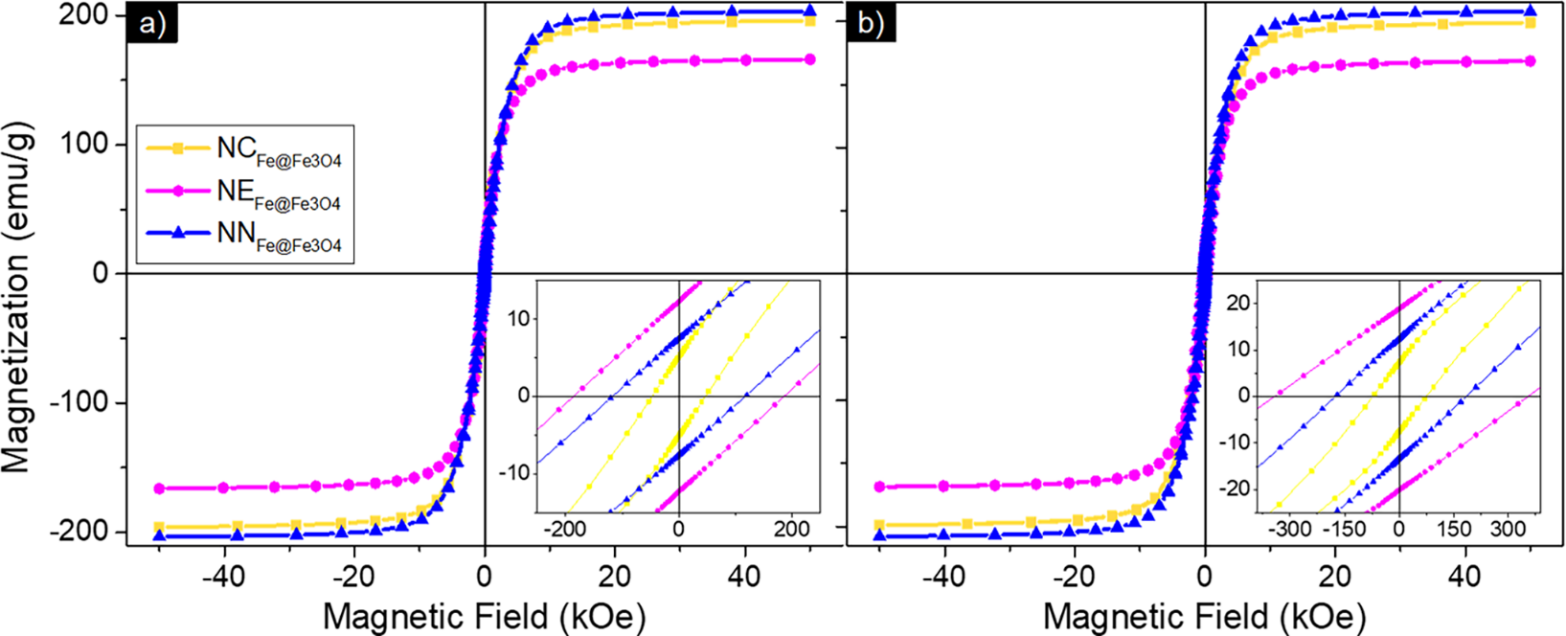
Magnetization as a function of the field of the iron-based Fe@Fe_3_O_4_ samples prepared in step 2 after the HyDR was recorded at (a) 300 K and (b) 5 K.

**Table 5:** Saturation Magnetization, Remanent Magnetization and Coercivity for All of the Iron-Based Fe@Fe_3_O_4_ Samples Prepared in Step 2 after the HyDR Extrapolated at 300 and 5 K

Sample	*M*_s_ (emu/g)	*M*_r_ (emu/g)	*H*_c_ (Oe)	Temperature (K)
${\mathrm{NC}}_{\mathrm{Fe}@{\mathrm{Fe}}_{3}O_{4}}$	195	5	48	300
	199	8	74	5
${\mathrm{NE}}_{\mathrm{Fe}@{\mathrm{Fe}}_{3}O_{4}}$	165	12	185	300
	168	19	350	5
${\mathrm{NN}}_{\mathrm{Fe}@{\mathrm{Fe}}_{3}O_{4}}$	203	8	117	300
	207	13	180	5

**Figure 10:**
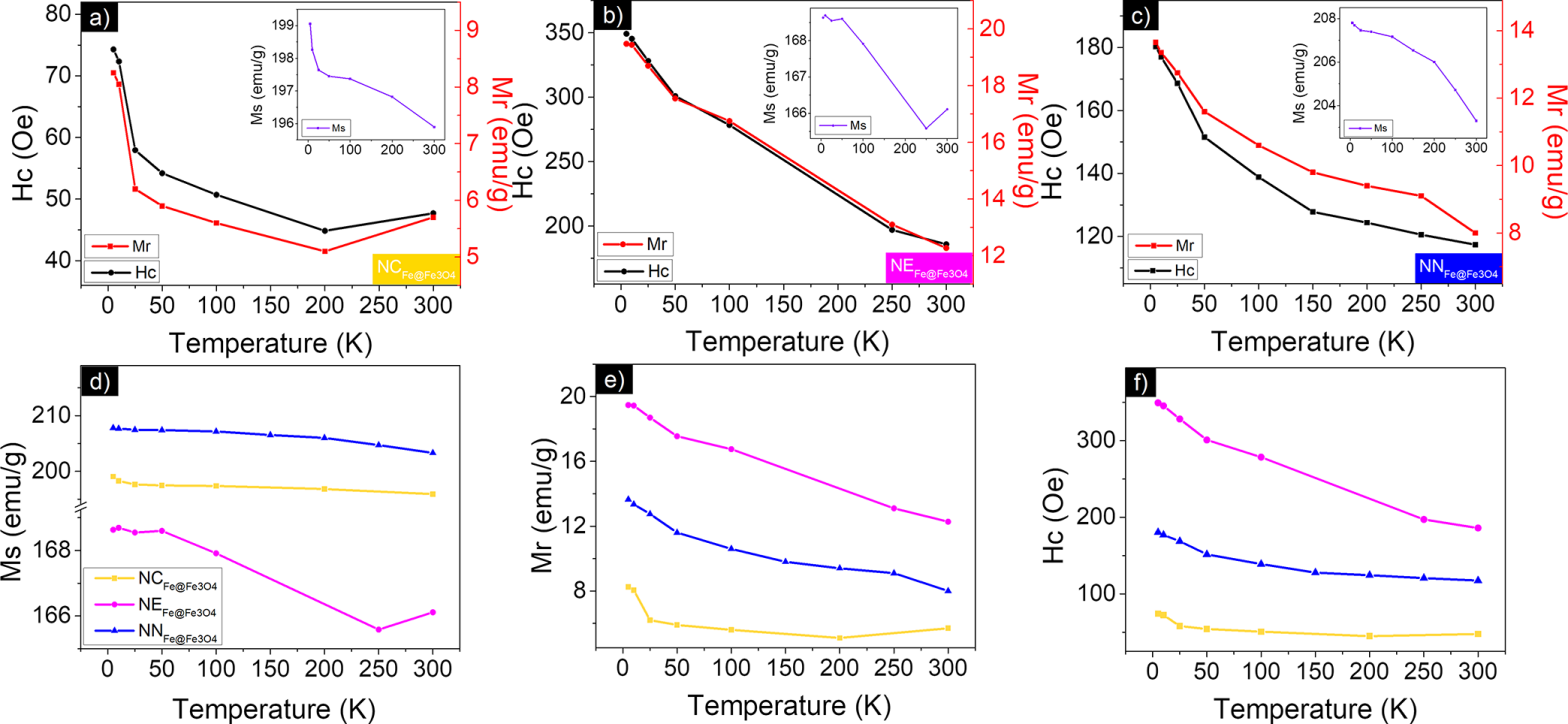
Saturation magnetization, Remanence, and coercivity of the iron-based Fe@Fe_3_O_4_ samples prepared in step 2 after the HyDR. (a) ${\mathrm{NC}}_{\mathrm{Fe}@{\mathrm{Fe}}_{3}O_{4}}$ , (b) ${\mathrm{NE}}_{\mathrm{Fe}@{\mathrm{Fe}}_{3}O_{4}}$ , and (c) ${\mathrm{NN}}_{\mathrm{Fe}@{\mathrm{Fe}}_{3}O_{4}}$ . Comparison between (d) saturation magnetization, (e) remanence, and (f) coercivity.

Figure 10a–c illustrates the behavior of coercivity, remanence, and saturation magnetization for samples ${\mathrm{NC}}_{\mathrm{Fe}@{\mathrm{Fe}}_{3}O_{4}}$ , ${\mathrm{NE}}_{\mathrm{Fe}@{\mathrm{Fe}}_{3}O_{4}}$ , and ${\mathrm{NN}}_{\mathrm{Fe}@{\mathrm{Fe}}_{3}O_{4}}$ , respectively, based on data extrapolated from hysteresis loops recorded at various temperatures (Table S2). In all samples, coercivity decreased with increasing temperature, showing a trend similar to that of remanent magnetization, which is typical for ferromagnetic or ferrimagnetic materials. A similar pattern was observed for the saturation magnetization across all samples.^9^

The high saturation magnetization in samples ${\mathrm{NC}}_{\mathrm{Fe}@{\mathrm{Fe}}_{3}O_{4}}$ and ${\mathrm{NN}}_{\mathrm{Fe}@{\mathrm{Fe}}_{3}O_{4}}$ , as illustrated in Figure 10d, is primarily due to their substantial iron content, around 80%, as determined by XRD and Mössbauer analysis (see Table 4), with values approaching that of bulk iron (218 emu/g^9^). The major conversion of hematite into iron after the reduction process of the samples ${\mathrm{NC}}_{\mathrm{Fe}@{\mathrm{Fe}}_{3}O_{4}}$ and ${\mathrm{NN}}_{\mathrm{Fe}@{\mathrm{Fe}}_{3}O_{4}}$ can be correlated to the crystallographic axis. Prior to reduction, samples ${\mathrm{NC}}_{\alpha -{\mathrm{Fe}}_{2}O_{3}}$ and ${\mathrm{NN}}_{\alpha -{\mathrm{Fe}}_{2}O_{3}}$ present a preferential crystallographic axis along the (104) direction, which is more intense when compared to the (110). On the other hand, as confirmed by XRD analysis, sample ${\mathrm{NE}}_{\alpha -{\mathrm{Fe}}_{2}O_{3}}$ shows an increase in the (110) direction when compared to samples ${\mathrm{NC}}_{\alpha -{\mathrm{Fe}}_{2}O_{3}}$ and ${\mathrm{NN}}_{\alpha -{\mathrm{Fe}}_{2}O_{3}}$ (Section 3.2.1), which leads to a slower conversion to iron.

Sample ${\mathrm{NN}}_{\mathrm{Fe}@{\mathrm{Fe}}_{3}O_{4}}$ , in particular, exhibits higher saturation magnetization compared to ${\mathrm{NC}}_{\mathrm{Fe}@{\mathrm{Fe}}_{3}O_{4}}$ , which can be attributed to three factors: first the higher iron content (98%), and also the larger particle size and increased shape anisotropy.^8,65^ These combined characteristics enhance the alignment of magnetic moments, contributing to the greater saturation magnetization observed in sample ${\mathrm{NN}}_{\mathrm{Fe}@{\mathrm{Fe}}_{3}O_{4}}$ .

In contrast, sample ${\mathrm{NE}}_{\mathrm{Fe}@{\mathrm{Fe}}_{3}O_{4}}$ displays reduced saturation magnetization due to its increased iron oxide content (magnetite), as confirmed by XRD and Mössbauer spectroscopy. Magnetite has a lower intrinsic magnetic moment per unit volume compared with pure iron, leading to a lower saturation magnetization (92 emu/g^9^). Consequently, as the proportion of magnetite increases, the overall magnetic moment per unit volume of the nanoparticles decreases because iron oxide contributes less to the total magnetic moment. This dilution effect reduces the saturation magnetization, highlighting that the size and shape of the particles influence the reduction process from hematite to nanostructured ferrite nanocomposites, with distinct mechanisms at play.

Additionally, the coercivity of sample ${\mathrm{NE}}_{\mathrm{Fe}@{\mathrm{Fe}}_{3}O_{4}}$ (Figure 10f) is significantly higher than that of samples ${\mathrm{NC}}_{\mathrm{Fe}@{\mathrm{Fe}}_{3}O_{4}}$ and ${\mathrm{NN}}_{\mathrm{Fe}@{\mathrm{Fe}}_{3}O_{4}}$ . As expected for ferromagnetic materials, an increase in *H*_c_ can be observed, whereas the Ms of the sample decreases, being in good agreement with the classical trend of *H*_c_ ∝ 1/*M*_s_.^9^ On the other hand, the higher coercivity of sample ${\mathrm{NN}}_{\mathrm{Fe}@{\mathrm{Fe}}_{3}O_{4}}$ can be explained by stronger anisotropic effects derived from particle shape, as these effects are more pronounced in needlelike nanoparticles with an aspect ratio (length/width) of 2.27 compared to ellipses-like (1.6) and cubic ones (1).^6^ In this case, the shape anisotropy effects seem to predominate over the increase in particle size, which would result in a decrease in coercivity, as reported for multidomain magnetic nanoparticles.^66^

Beyond coercivity, the remanent magnetization also increases with a higher iron oxide content $\left({\mathrm{NE}}_{\mathrm{Fe}@{\mathrm{Fe}}_{3}O_{4}}\right)$ due to enhanced anisotropy and domain wall pinning (Figure 10e). In this scenario, the magnetic moments within the oxide are less prone to relax back to a random state, leading to greater remanent magnetization after the external field is removed. By contrast, the higher remanence attributed to sample ${\mathrm{NN}}_{\mathrm{Fe}@{\mathrm{Fe}}_{3}O_{4}}$ can be derived from increased shape anisotropy.^6^

#### 3.3.2 Magnetization as a Function of Temperature (ZFC–FC)

From Figure 11a, it can be observed that the magnetization increases with increasing temperature for all the samples under zero-field-cooled (ZFC) and field-cooled (FC) conditions. The overall behavior indicates that the nanoparticles are in a magnetically blocked state, as confirmed by the M-H loops reported in the previous section and expected for ferromagnetic multidomain particles.^10^

**Figure 11:**
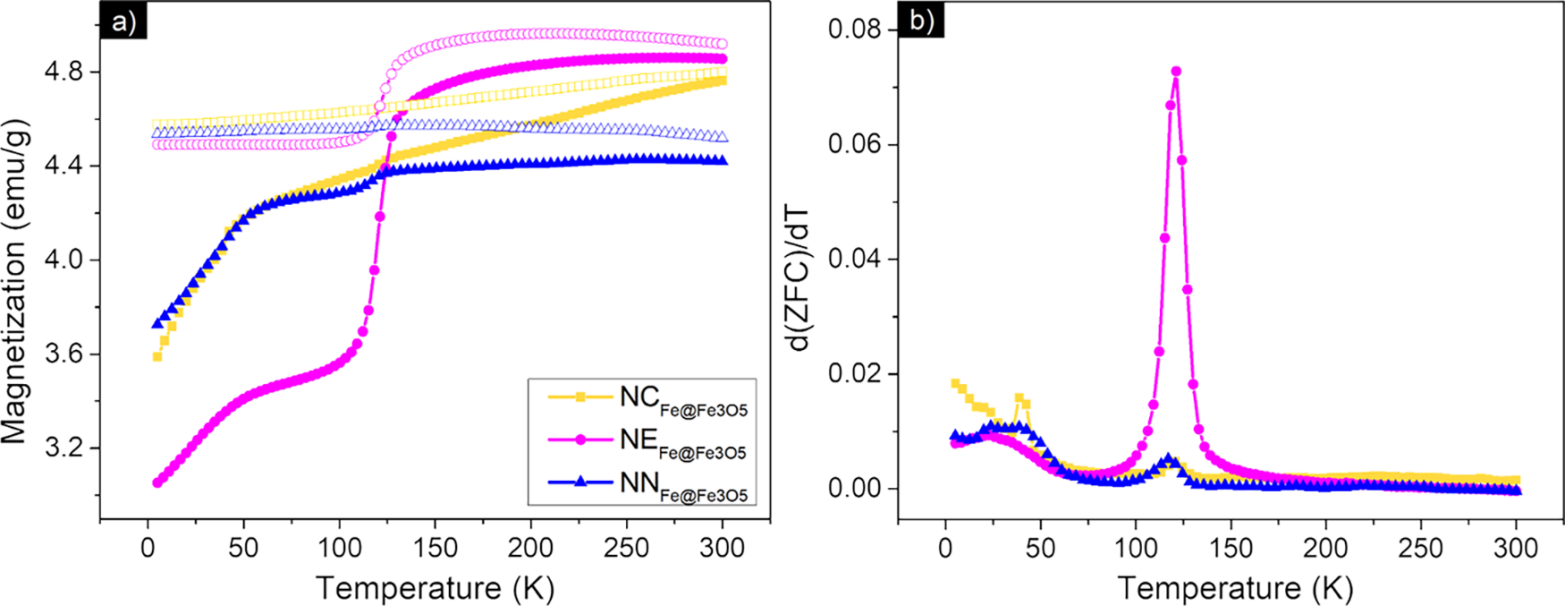
(a) ZFC–FC curves, (b) d­(ZFC)/d*T* of the iron-based Fe@Fe_3_O_4_ samples prepared in step 2 after the HyDR.

The well-defined shoulder observed for all the samples around *T*_v_ ∼ 120 K, extrapolated from the first derivative of the ZFC curve, is the signature of the characteristic Verwey transition of magnetite.^67^ As presented in Figure 11b in the d­(ZFC)/d*T*, all the samples presented a remarkable transition at 120.8, 121.4, and 117.6 K for samples ${\mathrm{NC}}_{\mathrm{Fe}@{\mathrm{Fe}}_{3}O_{4}}$ , ${\mathrm{NE}}_{\mathrm{Fe}@{\mathrm{Fe}}_{3}O_{4}}$ , and ${\mathrm{NN}}_{\mathrm{Fe}@{\mathrm{Fe}}_{3}O_{4}}$ , respectively, suggesting therefore the independence of the Verwey temperature from the particle shape.^6^ The higher intensity of the peak of sample ${\mathrm{NE}}_{\mathrm{Fe}@{\mathrm{Fe}}_{3}O_{4}}$ (corresponding to the sharp Verwey transition) can be attributed to the higher amount of magnetite, as confirmed by Mössbauer spectroscopy. Furthermore, although such transition is well-defined in bulk materials, it is often not observable in small magnetic nanoparticles in the superparamagnetic regime.^8,67^ Moreover, the sharp Verwey transition is a clear sign of highly crystalline and stoichiometric magnetite,^67^ suggesting that the reduction process promotes an improvement in the crystallinity of the magnetite phase.

### 3.4 Biocompatibility

To assess the biocompatibility of the fabricated nanoparticles for biomedical applications, the U-87 MG cell line was selected. As a result of this study (Figure 12), it could be concluded that for all the tested concentrations, the nanoparticles presented good biocompatibility, with their viability always being superior to 80%, making them promising nanoparticles to be used in the biomedical field. Moreover, studies with anisotropic magnetic nanoparticles have revealed that the large error bars associated with samples with aspect ratios, such as nanoellipses and nanoneedles, can arise from their partial agglomeration in the cell medium, affecting their interaction with the cells.^3^ The demonstrated biocompatibility provides valuable insight into the suitability of these nanoarchitectures for biomedical applications. Iron-based nanoparticles demonstrate immense potential to surpass iron oxide nanoparticles in applications like magnetic hyperthermia and MRI contrast agents due to their superior magnetic properties.^2,11^ The higher saturation magnetization of iron, 218 emu/g, compared to 92 emu/g for magnetite, can significantly enhance the performance of iron-based nanoparticles in both heating efficiency for magnetic hyperthermia and contrast enhancement for MRI. In hyperthermia, such nanoparticles generate heat more effectively under alternating magnetic fields due to their stronger magnetic moments and higher magnetic anisotropy, resulting in higher specific absorption rates (SAR).^5,68,69^ This allows for more effective cancer cell destruction using lower nanoparticle doses, minimizing side effects. In MRI, the strong local magnetic fields generated by these nanoparticles accelerate proton relaxation rates, particularly transverse relaxation (R_2_), leading to superior contrast in T_2_-weighted imaging and enabling a more accurate visualization of soft tissues. Moreover, anisotropic nanoparticles can further enhance the potential of iron-based nanoparticles for such applications.^5,10,68^ Although the use of iron-based nanoparticles, especially anisotropic ones, presents challenges, such as oxidation and stability in biological environments, the reported nanoparticles present the advantage of having a magnetite shell that prevents the oxidation of the iron core.

**Figure 12:**
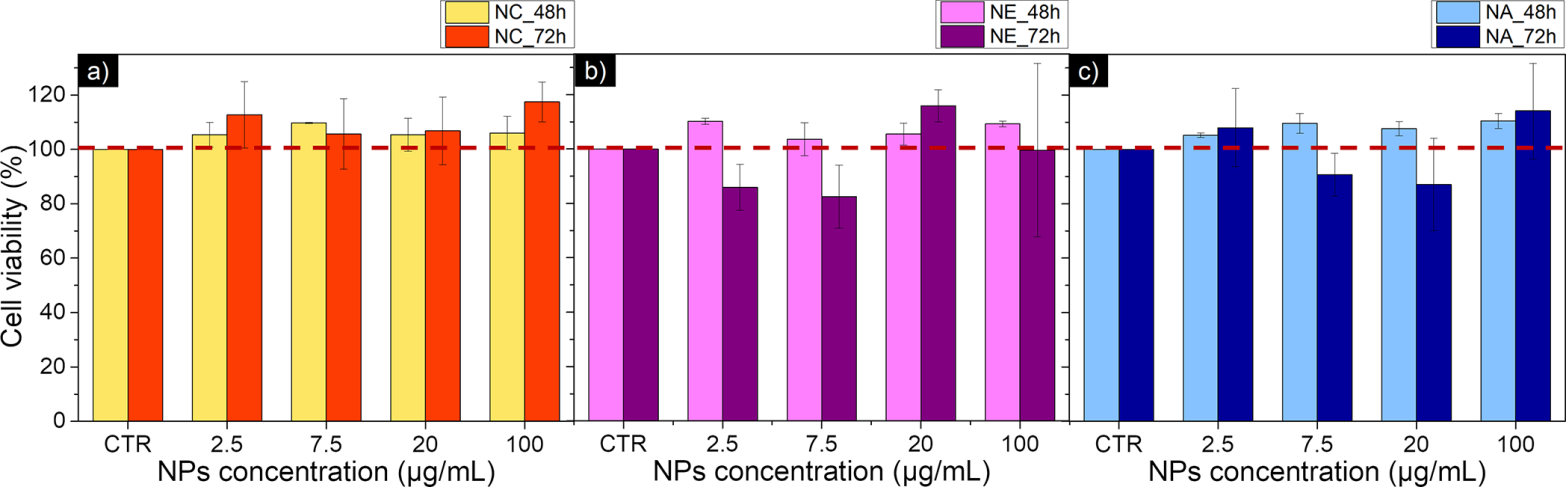
Viability studies of the iron-based Fe@Fe_3_O_4_ samples prepared in step 2 after the HyDR performed at 48 and 72 h postincubation in the U87 cells. Plot bars of (a) NC, (b) NE, and (c) NN.

## 4 Conclusions

This study highlights the successful synthesis and characterization of iron-based nanoparticles with distinct morphologies—nanocubes, nanoellipses, and nanoneedles—using a two-step process involving hydrothermal synthesis and HyDR. The morphology of the hematite precursors was controlled by varying the NH_4_H_2_PO_4_ concentration during synthesis, transitioning from nanocubes to elongated nanoellipses and nanoneedles. The reduction process preserved the overall morphology while reducing particle size and enhancing porosity, leading to the formation of a core–shell structure with a metallic iron core and a magnetite shell.

Structural and crystallographic analyses, including XRD and Mössbauer spectroscopy, confirmed the transformation of hematite into metallic iron and magnetite, with nanoneedles showing the highest iron content (97%). The Verwey transition observed in the ZFC–FC measurements confirmed that magnetite is the iron oxide phase present in the samples by the shape of the Verwey transition observed in all samples. Magnetic characterization as a function of the applied magnetic field demonstrated ferromagnetic behavior across all samples, with nanoneedles exhibiting the highest saturation magnetization due to their higher iron content, larger particle size, and enhanced shape anisotropy.

Biocompatibility tests confirmed that the nanoparticles maintain high cell viability (>80%) across all morphologies, making them suitable for biomedical applications such as magnetic hyperthermia and MRI contrast enhancement. Furthermore, this method allows fast and large-scale synthesis of particles, which is usually required for biomedical applications and many other applications.

## Supplementary Material



## Notes

Absorption/desorption isotherms plot for α-Fe_2_O_3_ nanoparticles fabricated in step 1 through hydrothermal synthesis (upper line) and Fe@Fe_3_O_4_ fabricated in step 2 after the HyDR (lower line) for NC, NE, and NN samples (Figure S1); parameters of the XPS fitting of the 2p spectra of samples Fe@Fe_3_O_4_ fabricated in step 2 after the HyDR (Table S1); *H*_c_, *M*_r_, and *M*_s_ values of all the Fe@Fe_3_O_4_ samples fabricated in step 2 after the HyDR for all the recorded temperatures (Table S2) (PDF)
