# Supplementary material for: Anisotropic Porous Iron-Based Nanoparticles through Two-Step Hydrothermal and Hydrogen-Based Reduction: Enhanced Magnetic Performance for Potential Biomedical Applications
Source: ACS Appl Mater Interfaces. 2025 Mar 7;17(11):16602–15. doi: 10.1021/acsami.4c21063 (PMC12258619; doi:10.1021/acsami.4c21063)
Supplement: Supplementary file 1 [file am4c21063_si_001.pdf]

Supporting Information

Anisotropic Porous Iron-Based Nanoparticles through two-step Hydrothermal and Hydrogen-based reduction: Enhanced Magnetic Performance for Potential Biomedical Applications

Sofia Caspani[1], Francisco Javier Fernández-Alonso[2,3], Sofia M. Gonçalves[1], Celia Martín-Morales[2], Belén Cortes-Llanos[3], Bruno J. C. Vieira[4], João Carlos Bentes Waerenborgh[4], Laura C. J. Pereira[4], Arlete Apolinario[1], João P. Araújo[1], Maria Victoria Gómez-Gaviro[5], Vicente Torres-Costa[2], Miguel Jose Manso Silván[2,3], Célia Tavares de Sousa[2,3]*

[1]IFIMUP - Departamento de Física e Astronomia da Faculdade Ciências da Universidade do Porto, Rua do Campo Alegre 1021 1055, 4169-007 Porto, Portugal

[2]Departamento de Física Aplicada, Universidad Autonoma de Madrid, Ciudad Universitaria de Cantoblanco, 28049 Madrid, Spain

[3]Centro de Microanálisis de Materiales, Universidad Autónoma de Madrid (UAM), Campus de Cantoblanco, 28049, Madrid, Spain

[4]Centro de Ciências e Tecnologias Nucleares, DECN, Instituto Superior Técnico, Universidade de Lisboa, 2695-066, Bobadela, LRS, Portugal

[5]IiSGM, Instituto de Investigación Sanitaria Gregorio Marañón, Madrid, Spain.

*Corresponding author: Célia Tavares Sousa (celia.tsousa@uam.es)

Table S1. Parameters of the XPS fitting of the 2p spectra of samples Fe@Fe_3O_4 fabricated in step 2 after the HyDR.

Sample	Components	Region	Pos. (eV)	FWHM	Distr.	%Area
NC	Fe^{2+}	2p (3/2)	778.2	1.4	GL(40)	13.5
	Fe^{3+}	2p (3/2)	776.5	2.1	GL(40)	35.21
	Fe(0)	2p (3/2)	780.2	1.3	LA (1.2,4.8,3)	7.05
	Fe^{2+}	2p (1/2)	764.6	1.9	GL(40)	4.4
	Fe^{3+}	2p (1/2)	762.9	2.1	GL(40)	17.73
	Fe(0)	2p (1/2)	766.6	1.1	LA (1.2,4.8,3)	3.47
	Satellite 2+	2p (1/2)	771.6	3.8	GL(40)	6.73
	Satellite 2+	2p (3/2)	758.6	2.2	GL(40)	1.93
	Satellite 3+	2p (1/2)	768.0	4	GL(40)	6.12
	Satellite 3+	2p (3/2)	754.9	5.3	GL(40)	3.67
NE	Fe^{2+}	2p (3/2)	778.2	1.4	GL(40)	6.02
	Fe^{3+}	2p (3/2)	776.5	2.1	GL(40)	42.72
	Fe(0)	2p (3/2)	780.0	1.3	LA (1.2,4.8,3)	2.51
	Fe^{2+}	2p (1/2)	764.6	1.9	GL(40)	3.39
	Fe^{3+}	2p (1/2)	762.9	2.1	GL(40)	19.87
	Fe(0)	2p (1/2)	766.7	1.1	LA (1.2,4.8,3)	1.23
	Satellite 2+	2p (1/2)	772	3.8	GL(40)	8.86
	Satellite 2+	2p (3/2)	758.6	2.2	GL(40)	2.46
	Satellite 3+	2p (1/2)	767.9	4	GL(40)	8.28
	Satellite 3+	2p (3/2)	754.8	5.3	GL(40)	4.66
	Fe^{2+}	2p (3/2)	778.2	1.4	GL(40)	6.58
	Fe^{3+}	2p (3/2)	776.5	2.1	GL(40)	46.79

	Fe(0)	2p (3/2)	780.0	1.2	LA (1.2,4.8,3)	3.01
	Fe^{2+}	2p (1/2)	764.6	1.9	GL(40)	2.48
	Fe^{3+}	2p (1/2)	762.9	2.1	GL(40)	20.94
	Fe(0)	2p (1/2)	766.7	1.3	LA (1.2,4.8,3)	1.48
NN	Satellite 2+	2p (1/2)	771.9	2.9	GL(40)	5.13
	Satellite 2+	2p (3/2)	758.6	2	GL(40)	1.67
	Satellite 3+	2p (1/2)	768	4	GL(40)	6.95
	Satellite 3+	2p (3/2)	754.9	5.3	GL(40)	12.94

Table S2. Hc, Mr, Ms values of all the Fe@Fe_3O_4 samples fabricated in step 2 after the HyDR for all the recorded temperatures.

Sample	T (K)	Hc_+(Oe)	Hc (Oe)	Hc (Oe)	Mr_+ (emu/g)	Mr_- (emu/g)	Mr (meu/g)	Ms (emu/g)
	300	46.9	-48.5	47.7	5.7	-5.7	5.7	195.9
	200	44.8	-44.9	44.85	5.1	-5.1	5.1	196.8
	100	50.6	-50.8	50.7	5.6	-5.6	5.6	197.4
	50	54.1	-54.3	54.2	5.9	-5.9	5.9	197.5
	25	58.1	-57.8	57.95	6.2	-6.2	6.2	197.6
NC	10	73.4	-71.3	72.35	7.9	-8.2	8.05	198.3
	5	74.3	-74.3	74.3	8.1	-8.4	8.25	199.1
	300	186	185.8	185.9	12.28	12.27	12.275	166.1
	250	196.8	197.4	197.1	13.1	13.1	13.1	165.6
	100	278.3	278.6	278.45	16.78	16.72	16.75	167.9
	50	303.5	298.3	300.9	17.4	17.7	17.55	168.6
	25	330.5	325.9	328.2	18.5	18.9	18.7	168.5
NE	10	350.5	340.2	345.35	18.98	19.9	19.44	168.7
	5	354.8	343.7	349.25	18.95	20	19.475	168.6

NN	300	117.3	117.4	117.35	8	8	8	203.3
	250	120.4	120.7	120.55	9.1	9.1	9.1	204.7
	200	124.3	124.4	124.35	9.4	9.4	9.4	206.0
	150	127.8	127.8	127.8	9.8	9.8	9.8	206.5
	100	138.7	139	138.85	10.6	10.6	10.6	207.1
	50	151.5	151.6	151.55	11.6	11.6	11.6	207.4
	25	169.6	167.7	168.65	12.7	12.8	12.75	207.4
	10	182.5	171.4	176.95	12.9	13.8	13.35	207.7
	5	184.7	175.9	180.3	13.2	14.1	13.65	207.8

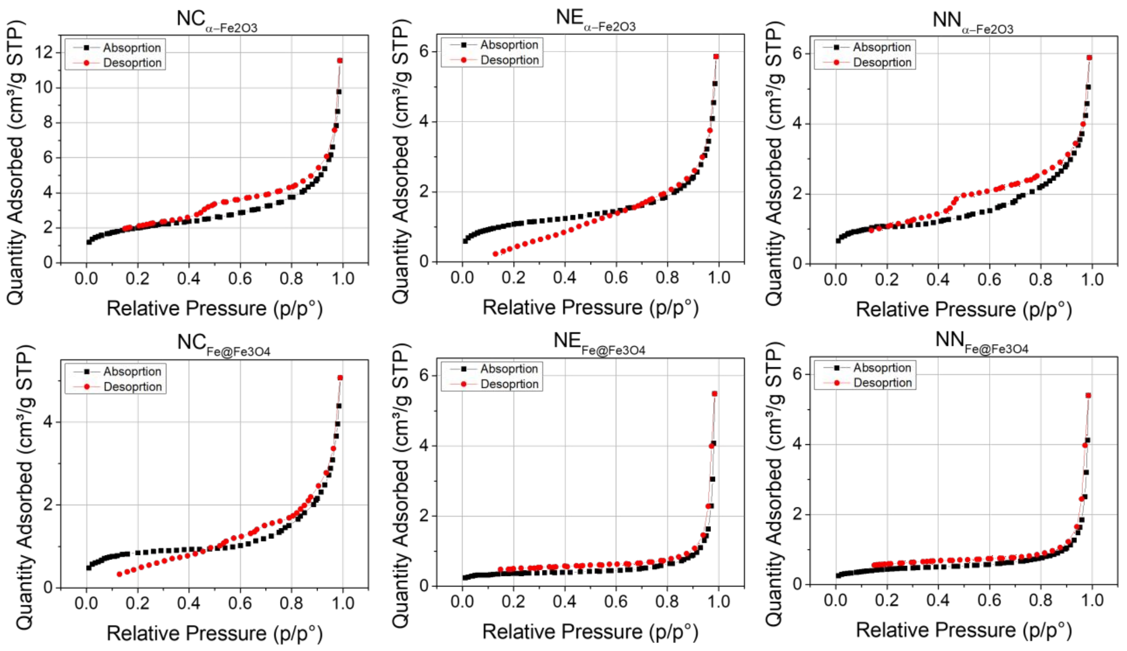

Figure S1. Absorption/Desorption isotherms plot for α-Fe2O3 nanoparticles fabricated in step 1 through hydrothermal synthesis (upper line) and Fe@Fe3O4 fabricated in step 2 after the HyDR (lower line) for NC, NE, NN samples.
